# The Vegetation–Climate System Complexity through Recurrence Analysis

**DOI:** 10.3390/e23050559

**Published:** 2021-04-30

**Authors:** Andrés F. Almeida-Ñauñay, Rosa María Benito, Miguel Quemada, Juan Carlos Losada, Ana M. Tarquis

**Affiliations:** 1 Centro de Estudios e Investigación para la Gestión de Riesgos Agrarios y Medioambientales (CEIGRAM), Escuela Técnica Superior de Ingeniería Agronómica Alimentaria y de Biosistemas (ETSIAAB), Universidad Politécnica de Madrid, Senda del Rey, 13, 28040 Madrid, Spain; miguel.quemada@upm.es (M.Q.); anamaria.tarquis@upm.es (A.M.T.); 2 Complex Systems Group, ETSIAAB, Universidad Politécnica de Madrid, Avda. Puerta de Hierro, no. 2, 28040 Madrid, Spain; rosamaria.benito@upm.es (R.M.B.); juancarlos.losada@upm.es (J.C.L.); 3 Department of Agricultural Production, ETSIAAB, Universidad Politécnica de Madrid, Avda. Puerta de Hierro, no. 2, 28040 Madrid, Spain; 4 Department of Applied Mathematics, ETSIAAB, Universidad Politécnica de Madrid, Avda. Puerta de Hierro, no. 2, 28040 Madrid, Spain

**Keywords:** cross-correlation, recurrence plots, vegetation indices, grasslands

## Abstract

Multiple studies revealed that pasture grasslands are a time-varying complex ecological system. Climate variables regulate vegetation growing, being precipitation and temperature the most critical driver factors. This work aims to assess the response of two different Vegetation Indices (VIs) to the temporal dynamics of temperature and precipitation in a semiarid area. Two Mediterranean grasslands zones situated in the center of Spain were selected to accomplish this goal. Correlations and cross-correlations between VI and each climatic variable were computed. Different lagged responses of each VIs series were detected, varying in zones, the year’s season, and the climatic variable. Recurrence Plots (RPs) and Cross Recurrence Plots (CRPs) analyses were applied to characterise and quantify the system’s complexity showed in the cross-correlation analysis. RPs pointed out that short-term predictability and high dimensionality of VIs series, as well as precipitation, characterised this dynamic. Meanwhile, temperature showed a more regular pattern and lower dimensionality. CRPs revealed that precipitation was a critical variable to distinguish between zones due to their complex pattern and influence on the soil’s water balance that the VI reflects. Overall, we prove RP and CRP’s potential as adequate tools for analysing vegetation dynamics characterised by complexity.

## 1. Introduction

Pasture grasslands are considered one of the most important ecological systems in the Iberian Peninsula, providing multiple agro-environmental services [1], including biodiversity and meat production. Several studies [2,3] presented pasture grasslands as a spatial temporal-varying complex ecological system involving multiple variables. Thus, historical time-series analysis [4] is considered an appropriate method to examine and characterise pasture grasslands complexity. In this work, we aim to assess the response of two different Vegetation Indices (VIs) time series to the temporal dynamics of temperature and precipitation in a semiarid area, characterised by a significant presence of bare soil and dead vegetation.

Multiple research studies [5,6] demonstrated that climate variables greatly influence vegetation growing. Among them, precipitation, and temperature [7] have been pointed out as the most direct driver factors for plant growth. Based on this assumption, Gesner et al. [8] showed that vegetation growth is strongly correlated to temporal and spatial patterns of precipitation in semiarid ecosystems, mainly because of the extreme precipitation events and seasonal shifts that affect vegetation dynamics. The temperature has also been pointed out as a critical driving factor in vegetation activity [9]. In this way, Piao et al. [10] reported that temperature showed positive effects on grasslands growth; decreasing, as temperature rises in high-cold areas. In the study area, two zones were selected to observe the interactions between climate and vegetation in two distinct semiarid zones. Both zones presented different precipitation regimes and annual temperatures. Ávila zone (ZAV) showed a lower annual mean precipitation and annual mean temperature in comparison to the Madrid zone (ZMA).

Researchers successfully applied remote sensing techniques in the agricultural [11,12] and environmental fields to monitor vegetation cover changes. Nowadays, satellites allow obtaining historical real-time acquisition data [13] about the vegetation status in vast areas. Optical remote sensing techniques allow tracking vegetation cover due to its specific spectral behaviour in the visible (0.4–0.7 mm) and infrared (0.74–1.11, 1.3–2.5 mm) bands of the electromagnetic spectrum. However, alternative approaches, such as radar techniques, have been pointed out as a promising tool for vegetation monitoring, crop mapping and soil moisture estimation. Synthetic aperture radar (SAR) based on techniques are the most widely used [14] in the agricultural field of study. Their principal advantages are the capacity to obtain information independent of the weather conditions, minimising the atmospheric effects, and the capacity to penetrate through the soil. However, they also present disadvantages compared to optical remote sensing techniques, such as possible speckle effects [15], that could reduce the quality of SAR imagery and the disturbance of topography [16] in hilly regions.

Vegetation indices (VIs) proved to be a powerful tool to characterise vegetation among all the optical remote sensing techniques. They are defined as a combination of two or more spectral bands related to vegetation status. It has been proved that VIs present a close relationship with climate [17] across different biomes and bioregions [18]. Significantly, the Normalised Difference Vegetation Index (NDVI) demonstrated an excellent indicator of the vegetation growth conditions [19,20] and the biophysical characteristics of ecosystems. Multiple studies revealed [21,22] that NDVI is an adequate tool to monitor rangelands conditions, being the most widely used spectral VI [23] by ecologists and agriculturalists until nowadays. Its efficiency is based on its capacity to reduce variability caused by the reflectance of the soil background [24], illumination, and view angle variation. NDVI values tend to increase during the growing season, showing the biomass increase due to the intense photosynthetic activity. On the contrary, there is a gradual reduction of NDVI values when there is a lack of water or the temperature is excessively higher.

Several authors [25,26,27] pointed out that differentiated bare soil and dead vegetation are still challenging due to the disturbances in the VIs sensitivity. Regions with sparse vegetation tend to generate high reflectance values that might saturate sensors or produce biased biomass and vegetation cover estimations. Thus, a modified soil-adjusted vegetation index (MSAVI) [28] was proposed as a solution to consider the soil background effect in semiarid areas. MSAVI has been successfully applied in numerous studies [29,30], especially in the estimation of above-ground biomass in semiarid areas.

Previous studies suggested that VIs response to climate differed at different timescales across the year’s seasons [31,32,33]. A high correlation between precipitation and NDVI has been reported at a yearly scale [6]. On the other hand, on a monthly scale, several studies revealed a delayed correlation between precipitation and NDVI [8,34]. Relationships between NDVI and temperature were also reported during specific periods. As an example, Tibetan Plateau (China) grasslands presented positive correlations during the growing season (May–September) [35]. Found correlations presented spatial–temporal variations [36], probably due to the differences in environmental factors or plant functional traits over which the correlations were calculated. In this way, researchers suggested that the optimal VI delayed response depends on the climate variable, shifting from one to two months in temperature and precipitation [37].

In ecological systems, temporal variability is frequently measured as the standard deviation of the records in a time series, though, these systems present nonlinear characters as in any complex system. In particular, VIs time-series present time cycles, allowing agro-environmental system dynamics description [38,39,40]. In this line, Eckmann et al. [41] introduced recurrence plots (RPs) as a simple way to envision the periodic or chaotic behaviour of a dynamical system through its phase space. Recurrence Plots–Recurrence Quantification Analysis (RP–RQA) are able to measure temporal determinism and predictability [42]. RPs are used to detect dynamical patterns in time series [43], and Recurrence Quantification Analysis (RQA) quantifies and characterises the small-scale structures in RP. RPs can be visually interpreted to distinguish non-stationary dynamics with either smooth or abrupt transitions [44] and finding the presence of periodic and non-periodic processes.

Furthermore, RPs can be extended to include multivariate relationships through Cross-Recurrence Plots (CRPs). CRP is defined as a bivariate extension of RP [43]. It is computed to analyse two variables by comparing their states and studying the dependencies between two different systems; it may be regarded as a nonlinear cross-correlation function. Multiple works analyse the behaviour of the VIs time series through RPs analysis. Li et al. [45] computed RPs to study the determinism and predictability of the NDVI series and its spatial patterns. Zurlini et al. [46] showed the landscape changes after a burn through RPs methodology on Enhanced Vegetation Index (EVI) time series. Semeraro et al. [47] showed the drought effects on a zone in the Amazon forest through RPs on MODIS EVI time-series.

Specifically, this study attempts to answer if: (i) VIs are an adequate tool to reflect the complexity of the relationships between vegetation and climate in grasslands, (ii) MSAVI performs better than NDVI to assess the vegetation response in a semiarid area and (iii) the recurrence plots methodology is a complementary tool to the study of the complexity of grassland ecosystems. This work is organised as follows: In Section 2, we present the study plots and the methods used to relate and compare the VIs response to climate in two different zones. In Section 3, we emphasise the main results of the paper. Then, in Section 4, we discuss the remarkable outcomes of the research. Finally, we present the key conclusions of the paper in Section 5.

## 2. Materials and Methods

### 2.1. Study Area and Plots Selection

**Figure 1:**
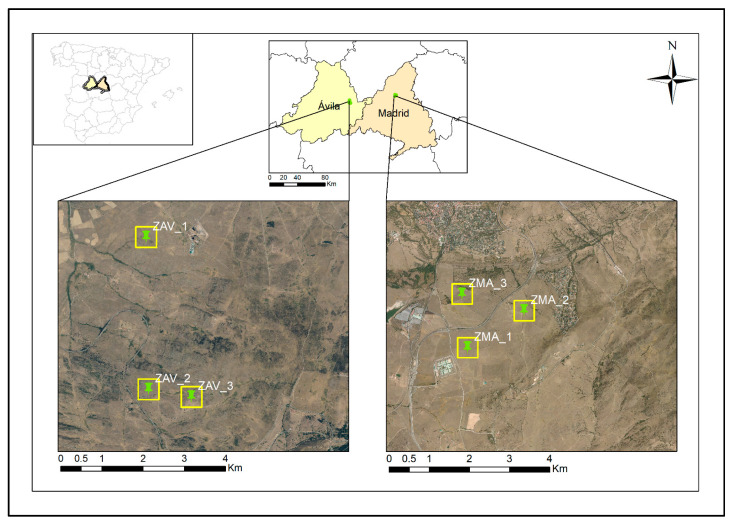
Localisation of the study zones and the three selected plots in each one. Left: ZAV, Tornadizos de Ávila, Ávila (ZAV_1, ZAV_2, and ZAV_3), Right: ZMA, Soto del Real, Madrid (ZMA_1, ZMA_2 and ZMA_3). Mosaic of the most current orthophotographs corresponding to sheet 0509 of the MTN50 in Spain, during the years 2017, 2018. Pixel size: 0.25 km. RGB composition.

We considered two study zones (Figure 1): ZAV situated in Tornadizos de Ávila, Ávila (Spain) and ZMA located in Soto Del Real, Madrid (Spain). Both sites are characteristics of inland Mediterranean grasslands under a Mediterranean climate with continental influence (temperate with a dry season and hot summer (Cfa), according to Köppen classification). Both grasslands rise during spring and autumn and have a summer senescence period and a vegetative winter dormancy of variable annual length (Table 1).

Plots were chosen based on three criteria: (i) maximum surface covered by pasture grassland without woodland, (ii) continuous pastureland practices during the analysed period, (iii) pastureland cover in the surrounding area. Thus, the ZAV zone was composed of three pixels of 500 × 500 m between (3°45′00′′ W, 3°46′00′′ W) and (40°43′00′′ N, 40°44′00′′ N). ZMA was composed of three pixels (500 × 500 m) enclosed between (4°32′00′′ W, 4°33′00′′ W) and (40°37′00′′, 40°39′00′′ N).

### 2.2. Acquisition of Satellite Data and VIs Calculations

Terra (EOS AM-1) satellite was launched in 1999, equipped with Moderate Resolution Imaging Spectroradiometer sensors (MODIS) that have been collecting reflectance data to date. MODIS imagery collection is structured in various products. Of them, the MOD09A1 was selected for this study. This product is a level-3 composite of the 500-m resolution, and the best pixel observation was chosen within eight days. The selection criteria for the best pixel observation were the aerosol content, view and solar zenith angle, cloud presence and clouds shadows [48,49].

Study plots reflectance was monitored from 2002 to 2018. Each year, 46 images were acquired, giving 782 images in the study period. In our case, only two of all the spectral bands were extracted from the imagery collection: band 1 (RED: 620–670 nm) and band 2 (NIR: 841–876 nm). An average of each band per zone was applied in the VIs calculation to assure a correct spectral characterisation.

Two VIs were calculated using the same spectral bands (RED and NIR), but a different approach in reducing the soil effect. The first one was the NDVI, calculated by the following Equation (1):

(1) $$\mathrm{NDVI}=\frac{\mathrm{NIR}-\mathrm{RED}}{\mathrm{NIR}+\mathrm{RED}}$$

where NIR is the reflectance in the near-infrared band (band 2) and RED (band 1) is the reflectance in the red band.

The second index was MSAVI, proposed as an improved version of NDVI. It includes bare soil and dead vegetation effect by adding a new variable named soil factor adjustment (*L_M_*). *L*_M_ is calculated by the following Formula (2):

(2) $$L_{M}=\frac{2*s*\left(\mathrm{NIR}-\mathrm{RED}\right)*\left(\mathrm{NIR}-s*\mathrm{RED}\right)}{\mathrm{NIR}+\mathrm{RED}}$$

where *s* is defined as the soil line given by a plot of RED vs. NIR brightness.

To obtain *s*, a set of points is extracted, characterised by the minimum NIR value within the RED breaks (0.005) in the RED vs. NIR plot [50]. A weighted least-squares linear regression is fitted over these points, being *s* the slope of the regression [51]. This method is recommended to be implemented when non-photosynthetic vegetation or bare soil is predominant in the scene, as it is our case in the Mediterranean summer.

Once the *s* parameter was calculated for each plot, the average s was used in Equation (2) to obtain the *L_M_* factor. Then, MSAVI was calculated by Equation (3):

(3) $$\mathrm{MSAVI}=\frac{\mathrm{NIR}-\mathrm{RED}}{\mathrm{NIR}+\mathrm{RED}+L_{M}}\times \left(1+L_{M}\right)$$

### 2.3. Meteorological Variables

Two different AEMET [52] stations were considered to provide daily measurements of average air temperature (Tmean, minimum temperature (Tmin), maximum temperature (Tmax) and precipitation. The meteorological station of ZAV is settled at the centre of Ávila (40°39′33.024′′ N, 4°40′48.000′′ W) and located at 1130 m.a.s.l. In the ZMA zone, the meteorological station is sited between Soto del Real and Colmenar Viejo (40°41′46.008′′ N, 3°45′54.019′′ W) at 1004 m.a.s.l).

### 2.4. Inter-Annual and Intra-Annual Analysis

On a first approach, we estimated the annual average of VIs, temperature, and accumulated precipitation. Then, a linear fitting was conducted to analyse the VIs and temperature annual trends. Precipitation was plotted to show the water availability along the time. A statistical test was performed to detect significant data trends [53]. The slope of the linear regression was compared to 0 using a Student t-test. If the t-estimated value was inferior to the critical t-value at the 95% level, then the slope was not significantly different from zero.

In the next step, a min–max normalisation (0–1) method was applied to determine whether annual climate variations were fluctuating with VIs behaviour. Each VI was compared to each climate variable in both zones (ZAV and ZMA).

A more in-depth analysis was performed to characterise date-to-date VIs behaviour. Average temperature and accumulated precipitation in an 8-day (PCP) period were estimated based on daily meteorological data. Descriptive statistics were applied to characterise each one of the 46 dates using box-plots charts. Based on the VIs trend changes in the date-to-date series, we distinguished several annual pasture phases. Individualised phase linear regressions were compared using the Chow test to confirm each phase datasets differences [54]. Then, linear regressions by phase were plotted to analyse the relationship between VIs and climate variables.

### 2.5. Time-Series and VIs Phase Cross-Correlations

Correlation Pearson’s coefficients (CR) were calculated to reveal relationships between VIs and climatic variables series along the time. Additionally, partial correlation coefficients (PCR) were calculated to detect the most determinant variable in the VIs response. Then, time-series lagged cross-correlation analysis was performed to obtain an optimal lag (*ℓ*) where the correlation between climate variable and VI is maximum. The following Formula (4) is applied:

(4) $$\rho \left(\ell \right)=\frac{cov\left(x\left(j,t\right),y\left(j,t-\ell \right)\right)}{\sigma_{x\left(j,t\right)}\sigma_{y\left(j,t-\ell \right)}}$$

where *x*(*j, t*) are the VI values, NDVI or MSAVI, at year *j* and time *t*. The *y*(*j,t* − *ℓ*) are the meteorological values, temperature, or precipitation, at year *j* and delayed *ℓ* times the lag time, which is eight days.

Pearson’s coefficients analysis was replicated in each VI phase, defined in Section 2.4. Then the expression becomes (5):

(5) $$\rho \left(i,\ell \right)=\frac{cov\left(x_{i}\left(j,t\right),y_{i}\left(j,t-\ell \right)\right)}{\sigma_{x_{i}\left(j,t\right)}\sigma_{y_{i}\left(j,t-\ell \right)}}$$

where *x_i_* (*j,t*) are the VI values at year *j* and time *t* that belong to phase *i*. The *y_i_*(*j,t* − *ℓ*) are the meteorological values at phase *i* at year *j* and delayed *ℓ* times the lag time, which is eight days.

### 2.6. Recurrence Plots and Recurrence Quantification Analysis

Recurrence plots (RP) allow visualising system states in the phase space. In complex dynamical systems, recurrence is related to the temporal evolution of dynamical systems trajectories in the phase space.

Generally, to compute an RP, an embedding dimension (m) and a time-delay (τ) are necessary. Delay, τ, is the minimum time lag to minimise the autocorrelation of a time series. Then, m represents the number of independent variables needed to characterise the dynamics of the system. Finally, RP is a square matrix, with time on both axes, of pairwise Euclidean distances between the reconstructed system states to which a distance threshold (ε) is applied [43]. Mathematically RP is defined as:

(6) $$R_{ij}=\Theta \left(\varepsilon -\|{\vec{x}}_{i}-{\vec{x}}_{j}\|\right),\;\;\;{\vec{x}}_{i}\in {\mathbb{R}}^{m},\;\;\;\;\;\;i,\;j=1\;...\;\;N,$$

where *N* is the number of measured states ${\vec{x}}_{i}$, Θ is the Heaviside step function (i.e., Θ(*x*) = 1, if $\|{\vec{x}}_{i}-{\vec{x}}_{j}\|\leq \varepsilon$, and Θ(*x*) = 0 otherwise), ‖·‖ is a norm, and ε is a threshold previously defined based on the time-series properties. In this study, the phase space trajectories are based on the Euclidean distance between ${\vec{x}}_{i}$ and ${\vec{x}}_{j}$ of the series. If $R_{ij}=1$ at a time (*i*, *j*), is marked as a black dot in the position (*i*, *j*). Otherwise, if $R_{ij}=0,$ the corresponding states will be represented as white dots.

The same principle is maintained in the cross-recurrence plot (CRP) methodology. However, in CRP, two different time series are analysed simultaneously, and black dots represent the co-occurrence of similar states between two time series. Mathematically, a CRP $\left(x_{1},\;x_{2},\cdots x_{i},\cdots x_{N}\right)$ and $\left(y_{1},\;y_{2},\cdots y_{j},\cdots y_{N}\right)$, is calculated by:

(7) $$C_{ij}=\Theta \left(r-\|{\vec{x}}_{i}-{\vec{y}}_{j}\|\right),\;{\vec{x}}_{i},\;{\vec{y}}_{j}\in {\mathbb{R}}^{m},\;\;\;\;\;\;i,\;j=1\;...\;\;N,$$

Several measures of complexity have been proposed to be quantified by the RQA, though, in this work, we focused on Determinism (DET), Average length of structures (LT), Shannon’s Entropy (ENT), Laminarity (LAM) and trapping time (TT), the extended formulas are added in the Appendix A**.** Furthermore, RQA was extended by computing the diagonal-wise recurrence quantification profile [55]. The recurrence rate around the line of coincidence (LOC) and the surroundings time lags was calculated to measure the two time-series coupling as a lag function. The maximum number of lags to be analysed was six, the same as the cross-correlation method.

CRQA R package [55,56] was used to construct RP, obtain RQA measures and compute diagonal-wise recurrence profile. First, the VIs series were normalised using a z-score normalisation; then, the distance matrix was rescaled based on the maximum value following the recommendations of Webber and Zbilut [57]. Optimizeparam function is then computed to find the three parameters’ optimal values (τ, m, and ε). The delay (τ) was found by obtaining the local minimum where mutual information drops to both series [58]. The embedding dimension (m) was calculated by the false nearest neighbours’ algorithm [59]. The threshold ε was estimated by an iterative process based on the time-series’ standard deviation (SD). In this work, ε was limited to 5% of the recurrence rate (RR) in all the cases. When multiples values of m, τ, were obtained by the optimisation, the maximum of them was selected as the optimal value to apply in the construction of CRPs.

The quantification of RP and CRPs structures was calculated with the Crqa function using the values obtained from the optimisation function. Then, the drpfromts function was computed to plot diagonal-wise recurrence profiles in the RPs and CRPs.

## 3. Results

### 3.1. Soil Line Acquisition

**Figure 2:**
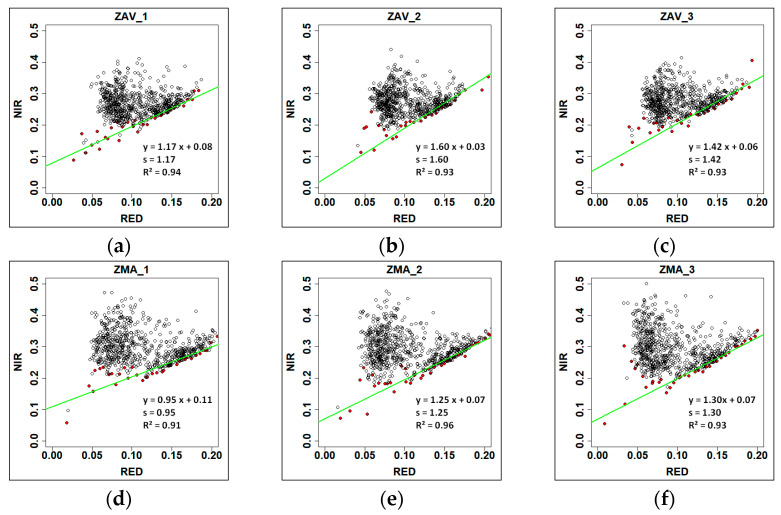
Soil slope (s) estimation from the spectral bands NIR vs. RED plots adapting the minimum NIR method [50]. Inside each figure, the regression equation was obtained from the Weighted Least Squares method. Each black point represents the NIR vs. RED reflectance in an 8-day interval between 2002 and 2018. Red points are the minimum value of NIR at 0.005 RED breaks. Panels (**a**–**c**) correspond respectively to the soil slope of pixels 1, 2 and 3 for the Tornadizos de Ávila (ZAV) zone. Panels (**d**–**f**) correspond respectively to the soil slope of pixels for the Soto del Real (ZMA) zone.

The soil line was calculated for each studied plot in each zone, and the results are displayed in (Figure 2). RED-NIR method’s linear regression displayed an R2 > 0.90 in all the cases. ZAV *s* values were higher than ZMA in most cases. We could not detect significant differences between the *s* values from ZAV and ZMA, even if the *s* average in ZAV (1.40) tends to be higher than in ZMA (1.17).

### 3.2. Inter-Annual Analysis

**Figure 3:**
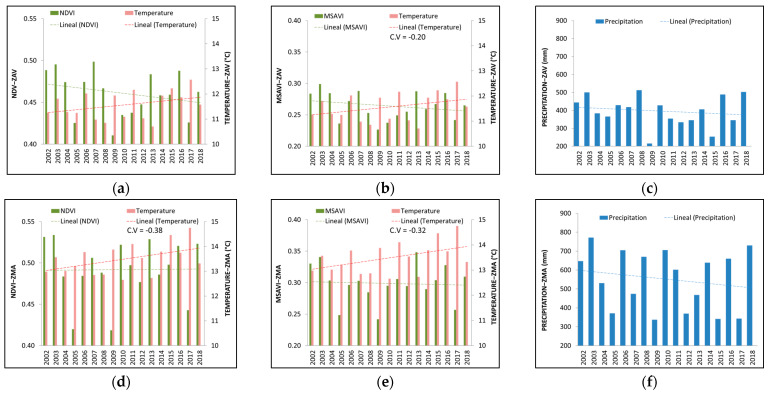
Inter-annual variations of vegetation indices (Normalised Difference Vegetation Index—NDVI, modified soil-adjusted vegetation index—MSAVI) and meteorological parameters of water (Annual accumulated precipitation) and energy (Annual average Temperature) from 2002 to 2018 in Tornadizos de Ávila (ZAV) (**a**–**c**) and Soto del Real (ZMA) (**d**–**f**)**.**

In the time series, VIs, temperature, and precipitation values were higher in ZMA than ZAV (Figure 3). VIs and precipitation displayed a descending trend over the years in both zones. In contrast, temperature showed an ascending trend in both zones. All the estimated slopes were non-statistically significant (Supplementary Material Table S1).

**Figure 4:**
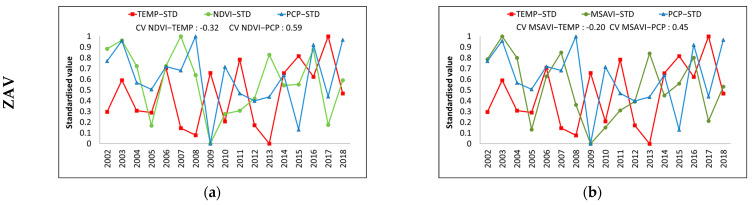

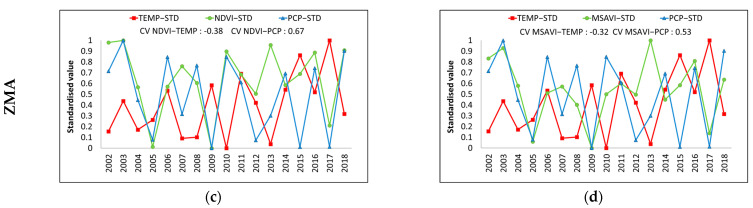
Relationship between the standardised NDVI and MSAVI with standardised climate parameters of energy (Annual average Temperature) and water (Annual accumulated precipitation) in Tornadizos de Ávila (ZAV) (**a**,**b**) and Soto del Real (ZMA) (**c**,**d**) at a yearly scale from 2002 to 2018.

Generally, an inverse relationship was observed between VIs and temperature fluctuations (Figure 4). For instance, in 2009 and 2017, the temperature was remarkably higher in both zones; consequently, NDVI and MSAVI showed a severe fall. In contrast, we detected a direct relationship between VIs and precipitation. Usually, when precipitation was limited, VIs tended to be reduced. This tendency was observed over 2009 and 2017 in ZAV, and over 2003–2004 in ZMA, respectively.

In general, we detected that both VIs showed similar inter-annual variations in both zones. However, there were unusual VIs changes in certain years, i.e., in 2007, VIs showed a remarkable rise, even when temperature and precipitation did not show a notable change of trend.

### 3.3. Intra-Annual Analysis

**Figure 5:**
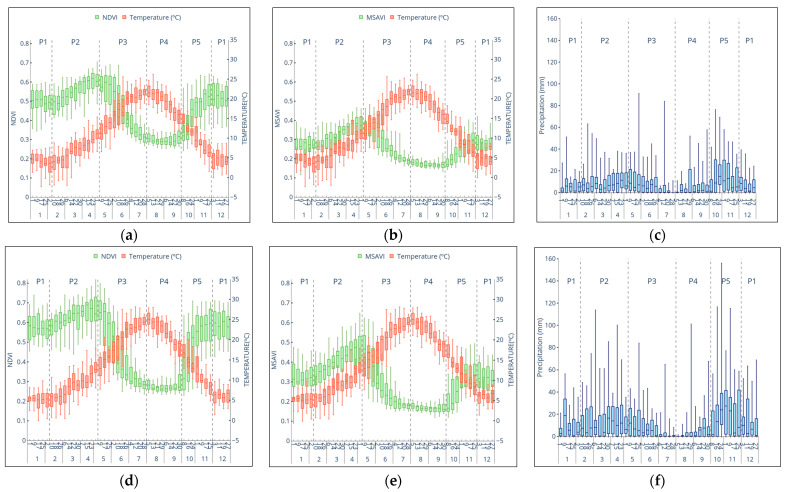
Boxplots of vegetation indices and meteorological parameters of energy (Average temperature) and water (Accumulated precipitation) in 8 days periods from 2002 to 2018 in Tornadizos de Ávila (ZAV) (**a**–**c**) and Soto del Real (ZMA) (**d**–**f**).

The VIs phases were closely related to the Mediterranean climate seasons (Table 2), being (P1 and P2) the cold season, (P4) the hot season and (P3 and P5) the transitional periods. Chow’s test revealed that all of them were significantly different from each other (Supplementary Material Table S2). We found that VIs, temperature, and precipitation were higher in ZMA than ZAV on an intra-annual scale (Figure 5). We also observed that VIs and precipitation were notably different between zones in P2 and at the beginning of P3 and P5. It should be noted that ZMA VIs declined faster in P3 and increased quicker in P5 than ZAV VIs. NDVI dispersion was generally greater than MSAVI. At the same time, VIs and precipitation showed a higher data dispersion in ZMA than ZAV. Both variables reached their dispersion peak in the same period of the year (P3 and P5).

**Figure 6:**
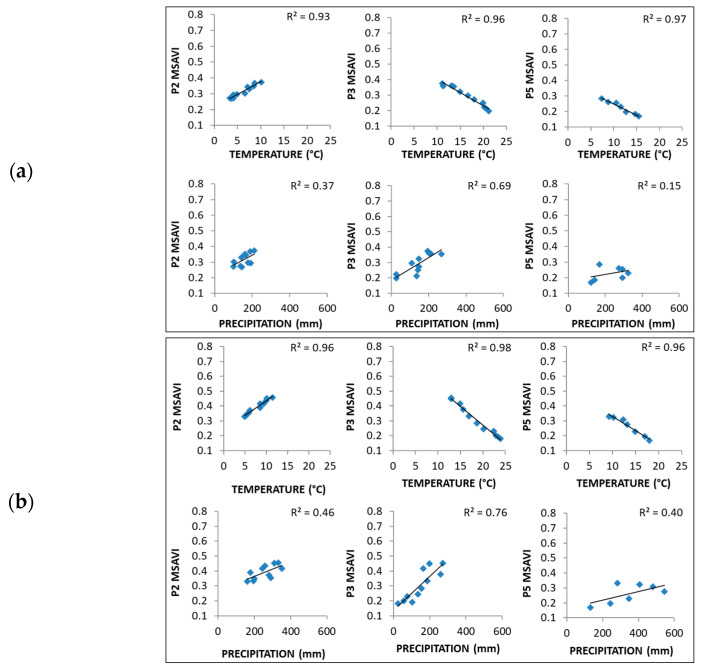
Growing phases response of MSAVI and water (Accumulated precipitation) and energy (Average temperature) parameters, in 8 days periodsat Tornadizos de Ávila (ZAV) (**a**) and Soto del Real (ZMA) (**b**). In each figure, the regression equation was obtained from the Least Squares method.

Based on the box plots results, it was observed that P1 and P4 phases did not show any variation along the time, being stable and less dispersed than the other phases. For this purpose, linear regression analysis between VIs and climate variables was conducted only in the phases in which a trend was observed in VIs (Figure 6 and Supplementary Material Figure S1). In this way, the most critical vegetation–climate driving factors were detected. Generally, the temperature was identified as the potential driving factor in the vegetation–climate system as it showed R^2^ > 0.9 in all the studied phases in both zones. We observed that the temperature trend varied throughout the year, being positive in P2 and negative in P3. Instead, precipitation showed lower R^2^ values than temperature, being the highest (>0.7) in P3, and maintained the same positive trend in all the phases. From this point, we detected that both indices showed similar behaviour, though, generally, MSAVI showed better results, suggesting that its dynamics would be more distinct than NDVI. Thus, NDVI analysis and results are presented in the Supplementary Material.

### 3.4. Time-Series Correlation

Pearson’s coefficients analysis revealed that all correlation coefficients between VIs and meteorological time-series were statistically significant along the time (Table 3 and Supplementary Material Table S3). The corresponding Pearson’s coefficients for temperature showed a negative relationship, whereas precipitation displayed a positive relationship. The temperature was the most correlated variable, and partial coefficients indicated that temperature is the main driving factor in the relationships between climate variables and VIs.

**Figure 7:**
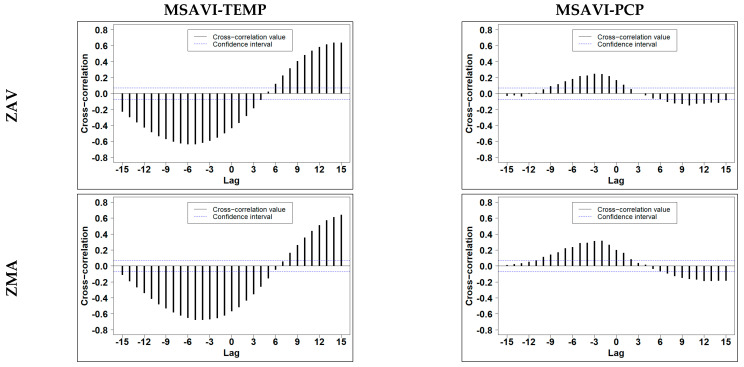
Time series cross-correlation between MSAVI and climatic variables time series, Temperature (TEMP) and Accumulated precipitation (PCP) in Tornadizos de Ávila (ZAV) and Soto del Real (ZMA). Each lag is of an 8-days period; the blue dot line is the confidence interval at 95%.

Cross-correlation showed the sinusoidal behaviour of the climate variables and the seasonality of VIs (Figure 7 and Supplementary Material Figure S2). The Lagτ varied between variables and zones, i.e., MSAVI-PCP showed an Lag of −2 (16 days) in ZAV, while Lag was of −3 (24 days) in ZMA. The Lag also fluctuated depending on the VI used, NDVI could not differentiate precipitation lags between zones; meanwhile, MSAVI distinguished them.

### 3.5. Correlation by Phase

Pearson correlation coefficients by phases (Table 4 and Supplementary Material Table S4) indicated that VIs dynamics varied over a year. Correlation coefficients varied between zones, being the highest difference in P3 and P5. The temperature was the most correlated climate variable, achieving values higher than 0.7 in P3 and P5 in both zones and VIs. As expected, there were some inaccuracies in the precipitation; thus, it was not possible to obtain high correlations values (<0.5). However, in P3, MSAVI showed significant results (0.270 in ZAV and 0.248 in ZMA), most likely due to the decrease of MSAVI data dispersion during the dry season.

A fluctuating lag was observed in cross-correlation coefficients by phases, pointing out the different VIs dynamics during a year (Table 5 and Supplementary Material Table S5). The most correlated variable was the temperature. Precipitation did not achieve higher correlation values, though a lag was needed in all the cases. As a result, the correlation by phase method improved the correlations in all the precipitation cases. Overall, cross-correlation by phases was significant in P3 and P5, allowing us to achieve better correlation coefficients than time-series analysis, regardless of VI and zone.

In NDVI and temperature, *ℓ* varied in both zones from zero in P2 to 5 (40 days) in P5. In the MSAVI case, temperature *ℓ* showed a similar pattern to the NDVI case, varying only in the P5 phase on ZMA. Concerning precipitation, in the NDVI case, an *ℓ* of 2 (16 days) was found in all the phases studied for both zones. Though, MSAVI showed different precipitation *ℓ* depending on the zone and the phases. Precipitation *ℓ* was 3 (24 days) in the phases when the VI increases (P2 and P5) in both zones. In P3, with a decreasing trend in the VI, the precipitation *ℓ* was 2 in Avila and 1 in Madrid. This fact pointed out a higher precipitation MSAVI sensitivity and recognised *ℓ* as a variable able to distinguish different zones in the same phase.

MSAVI performance was better than NDVI in winter and at the beginning of the spring (P2). It was remarkable because precipitation dispersion reached its peak during this phase. This fact might reveal that MSAVI might improve NDVI results in the springtime at a correct time scale. It is recommendable to analyse both series from a dynamic point of view, emphasising the behaviours of MSAVI in both zones.

### 3.6. RPs Characterisation and Recurrence Diagonal Profile

The Optimizeparam function was computed to estimate the parameters of RPs and showed that the embedding dimension (m) increased in all VI series for both zones (Table 6 and Supplementary Material Table S6). In the case of NDVI, m was 2 for both zones, then for the MSAVI case, m was 6 for ZAV and 8 for ZMA. The τ ranged from 8 to 11 varying between zones. The same dimensionality increase was detected in the climate variables where precipitation showed a higher dimension than the temperature in ZMA, pointing out a higher precipitation complexity than temperature.

**Figure 8:**
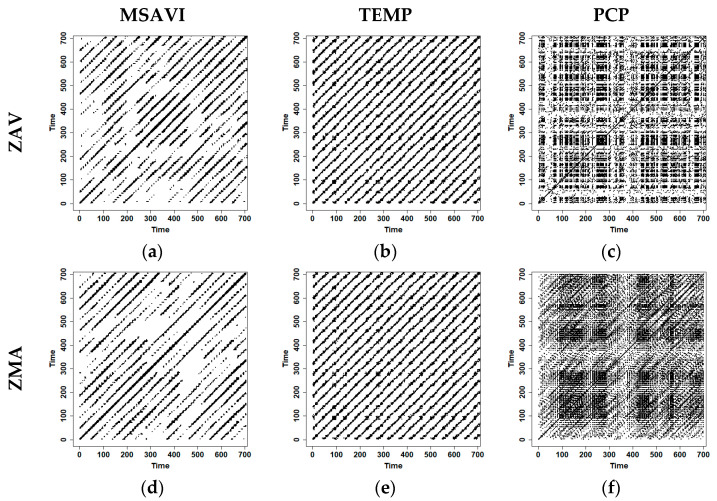
Optimised recurrence plots (RP) using normalised vegetation indices (MSAVI), Average temperature (TEMP) and Accumulated precipitation (PCP) data and rescaled distance matrix for Tornadizos de Ávila (ZAV) and Soto del Real (ZMA). Time units are represented as the X and Y-axis. Each time-unit is 8-days, coincident with 8-day composed MODIS images during the study period (2002–2018). Panels (**a**–**c**) correspond respectively to RP of MSAVI, Temperature and Accumulated Precipitation for the ZAV zone. Panels (**d**–**f**) correspond respectively to MSAVI, Temperature and Accumulated precipitation for the ZMA zone.

NDVI RPs showed a noisy behaviour, characterised by many isolated points. Meanwhile, MSAVI RPs showed white stripes on a large scale (Figure 8 and Supplementary Material Figure S3). Furthermore, we could observe that MSAVI RPs present small-scale structures and periodic patterns (diagonal line like-shapes). This kind of structure is visible in the temperature RP, where we could observe the temperature seasonality through diagonal-like structures. At the visual inspection, we did not detect significant temperature changes between the two zones.

In contrast, precipitation RP showed a distinct pattern in each zone. In ZAV, we could observe a block-like structure, whereas, in ZMA, we could distinguish a more line-like pattern. This difference was likely due to the different precipitation regimes in each zone.

**Figure 9:**
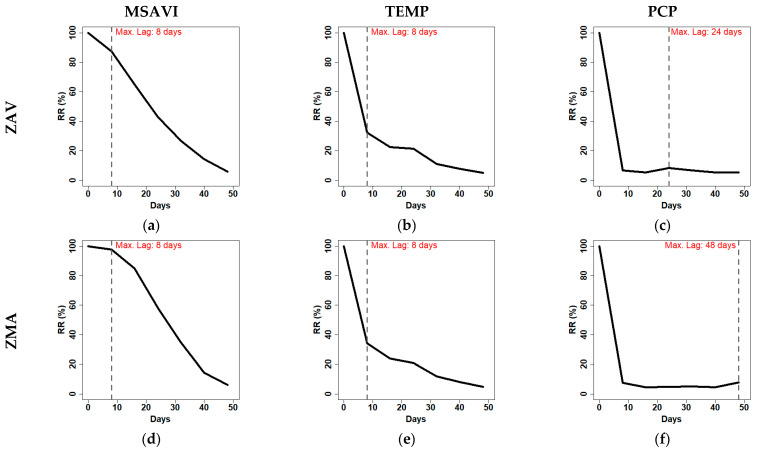
Diagonal-wise recurrence profile of the RPs obtained from the MSAVI, Average temperature (TEMP) and accumulated precipitation (PCP) in Tornadizos de Ávila (ZAV) (**a–c**) and Soto del Real (ZMA) (**d–f**).

We found that the profile tendency varied between VIs and zones (Figure 9 and Supplementary Material Figure S4). NDVI showed a more distinctive RR drop on the first days (0–8 days) in both zones. In contrast, MSAVI maintained higher values of RR until 16 days. As expected, temperature showed similar behaviour in the two zones. Meanwhile, PCP showed a different maximum lag. Both PCP profiles showed lower values of RR from eight days till the end. We speculate that different maximum lags are a consequence of the different PCP distribution in both zones.

### 3.7. CRPs Characterisation and CRPs Diagonal Profile

Once RPs were constructed, the maximum m and τ for the two time series were selected as the parameters to the construction of CRPS (Table 7 and Supplementary Material Table S6), then a RR of 5% was selected for all of them.

**Figure 10:**
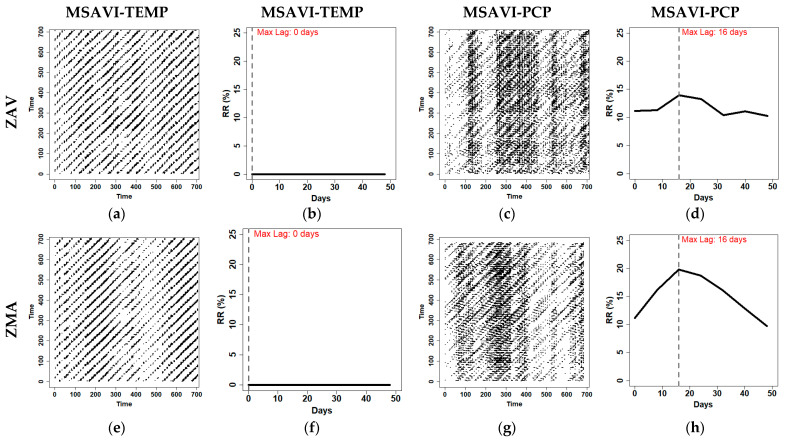
Optimised Cross-Recurrence Plots (CRPs) and diagonal-wise recurrence profiles between vegetation index data (MSAVI) temperature data (TEMP) and accumulated precipitation data (PCP) for Tornadizos de Ávila (ZAV) and Soto del Real (ZMA). Time units are represented as the X and Y-axis. Each time-unit is 8-days, coincident with 8-day composed MODIS images during the study period (2002–2018) in the CRPS. Lags are represented in days in the diagonal-wise recurrence profile The panels (**a**,**c**,**e**,**g**) represent the CRPs of MSAVI-TEMP and MSAVI-PCP. The panels (**b**,**d**,**f**,**h**) represent the diagonal-wise profile of the CRPS, respectively.

We detected the seasonal temperature effect on the VIs dynamics in the CRPs (Figure 10 and Supplementary Material Figure S5). In the case of NDVI-TEMP, we could not distinguish between zones. However, MSAVI-TEMP showed a different pattern in each zone, probably due to the distinct MSAVI dynamics that distinguish between different CRP patterns. Cross-recurrence profile allowed us to distinguish the interactions between VIs and climate variables in the LOC and the surroundings lags. The temperature did not show a difference between zones. As we observed in TEMP-VIs CRPs, the LOC was non-existent (Figure 10 and Supplementary Material Figure S5), and the surrounding regions were similar. Then it was expected that RR was near zero on the first lags. We believe that this fact was due to the temperature seasonality not being detected on the first lags.

In contrast to the temperature, CRPs of precipitation and VIs were able to characterise a different dynamic in each zone. In this case, we observed that NDVI-PCP CRPs showed vertical lines in ZAV and diagonal-like structures in ZMA. In MSAVI-PCP CRPs, the most distinct region zone occurred during 400–600 time-units, where ZMA presented an isolated point structure, while ZAV did not show any recurrence in that timeframe. This fact might be explained due to the increase of dimensionality produced by the precipitation in the CRPs. VIs-PCP recurrence profile showed an evident maximum lag of 16 days in the ZMA. In contrast, the ZAV presented a lower RR in precipitation, and the maximum lag was not evident, presenting a more stable recurrence profile. This difference can be explained because the CRQA analysis detected a higher number of precipitation events coupled to the MSAVI index, showing a more evident maximum lag. in the ZMA zone.

### 3.8. Recurrence Quantification Analysis of RPs and CRPs

We will present a more quantitative analysis by carrying out the RQA of the RPs and CRPs considered for each study zone (Table 8 and Supplementary Material Table S6).

Now, we present the values of DET, LT, ENTR, LAM and TT. The DET is related to the system’s random or periodic behaviour based on the density of recurrence points, being higher when the system shows more periodical behaviour. The MSAVI presented a higher DET in both zones, being ZMA the highest. DET obtained in precipitation RP was higher in ZAV than ZMA.

Concerning CRPs, MSAVI-TEMP showed a higher DET than NDVI-TEMP. Both of them showed a higher DET than PCP CRPs. MSAVI-PCP showed a DET increase in comparison to NDVI-PCP in ZMA. We believe that MSAVI could characterise better precipitation data dispersion, allowing us to improve the NDVI results in ZMA.

The LT is interpreted as the system’s predictability time, increasing when the predictability time is longer. The LT values in both VIs obtained were low. Temperature showed a higher LT than precipitation, suggesting that temperature predictability time was higher than precipitation. In CRPs, we observed similar results, being the LT of VIs-TEMP higher than VIs-PCP.

The ENTR value refers to the disorder of the system. MSAVI RPs showed a higher value than NDVI, higher in ZMA than ZAV. Concerning climate variables, temperature showed a higher ENTR than precipitation in their separate RPs and their CRPs with VIs.

The LAM value refers to the chaos–chaos transitions and is directly related to the detection of laminar states. The MSAVI LAM was higher than NDVI. Generally, temperature showed higher LAM values than precipitation. We also noticed that when precipitation was involved, LAM values tended to decrease in the CRPs, being temperature the highest in both cases.

The TT represents the average length of vertical structures and indicates how long the state will be trapped at the same time. The MSAVI showed a higher value of TT in both zones. In the climate variables RPs, Temperature TT was higher than precipitation TT. The same phenomenon happened in the CRPs, where TT was higher in VIs-TEMP than VIs-PCP.

## 4. Discussion

Concerning *s* values, we obtained different values of *s* in each zone. According to [60]’s findings, *s* increased when soil moisture grew. As shown in Table 1, ZAV topsoil was more clayey and less sandy than ZMA soil. Thus, a higher water holding capacity (WHC) was expected in ZAV than ZMA, explaining the differences between zones. We could not obtain significant differences between the *s* values from ZAV and ZMA, most likely because of the litter and non-photosynthetic material influence [61].

Temperature and precipitation yearly tendencies found in this work are consistent with what was reported in previous studies, where the temperature is increasing, and precipitation is decreasing in semiarid zones due to the climate change effect [62,63]. We detected specific years when VIs dramatically dropped (2005, 2009, and 2017). These years coincide with drought periods [64,65] that happened in Spain (2004–2008 and 2016–2017). These phenomena most likely negatively affected the vegetation growth; thus, VIs values decreased during this time. We expected a non-significant result in the trend slope because environmental works suggested a significant data quota is needed to obtain trustworthy climate trends at a yearly scale [66].

We obtained different inter-annual trends depending on the climate variable. An inverse relationship between temperature and VIs was found, in agreement with previous research [67], pointing out that NDVI and other optical indices are generally inversely related to temperature. In contrast, the precipitation was directly related to VIs. This fact ties nicely with previous studies [68] wherein precipitation events were related to vegetation growth, leading to increased VIs values.

Both VIs used the same NIR-RED spectral bands, leading to a similar performance [11]. However, the soil factor’s addition in the MSAVI case was expected to increase the sensitivity in semiarid areas [27,28,69]. In our results, MSAVI showed lower dispersion than NDVI, pointing out a better potential for characterisation of semiarid pasture grasslands.

Several studies [62,63,70] emphasise that precipitation and temperature combine in a dynamic and complex system; thus, their networks must be considered. As was reported by Suzuki et al. [71], NDVI could be affected by other complementary variables, such as evapotranspiration, that depends on the combination of local wetness and warmth. The lack of these variables might explain the unusual behaviours in the VIs time series that are not directly related to temperature or precipitation.

We believe that ZMA VIs were higher than ZAV because of the higher amount of precipitation in ZMA during P2 and at the beginning of the P5 phase. Precipitation events increased soil moisture leading to an increment in vegetation growth that VIs detected. We speculate that the interaction between soil moisture and soil texture with the vegetation in P2 and P5 might explain the differences between zones [72]. At the end of the rainy season (P2), both soils’ water storage is likely to be highest after the winter water recharge. However, water holding capacity was lower for the sandy than for the clayey soil (Table 1), and so less water was available in the ZMA than in the ZVA soil. During P3, the temperature raised, then the pasture in the ZMA depleted the soil water more quickly than in the ZAV. Therefore, ZMA vegetation decreased faster than ZAV (Figure 5). A similar effect took place at the beginning of the P5. Water storage in both soils was expected to be the lowest due to the previous dry season (P4). During P5, precipitation increased in both zones. ZMA sandy soil permeability was higher than ZAV clayey soil allowing a faster increment in ZMA vegetation than ZAV.

We detected an increase in the VIs dispersion during P3 and P5. This variability increase was expected due to the precipitation variability increase that occurred during spring and autumn. As reported by Grant et al. [73], precipitation variability leads to increased soil moisture variability. Therefore, grassland productivity is altered due to water availability fluctuations, leading to higher variability in VIs results.

Fu and Burgher [74] pointed out the temperature as the most potential driving factor in NDVI dynamics. They also revealed that temperature harms NDVI as we detected the same result in P3. This effect is explained by the limitation in vegetation growth produced by higher temperatures and fewer precipitations during the dry season. These climate conditions enhance the intensity of transpiration and reduce the available soil water [17]; thus, vegetation growth is expected to be limited in these unfavourable conditions.

Precipitation was the only variable that maintained the same positive trend in all the phases, pointing out that precipitation is regularly favourable in semiarid grassland growth. The same conclusion was achieved by Sala et al. [75], which presented a positive relationship between precipitation and pasture grasslands growth because of the soil moisture’s positive role in biomass production.

Multiple works have demonstrated transparent relationships between climate and VIs response [8,76]. Our results agree with [9], who showed a negative relationship between temperature and NDVI. In line with Liu et al. [68], we also found a positive correlation between precipitation and VIs in the semiarid area pasture grasslands.

Cross-correlation results allowed to expose the seasonal behaviour of the VIs over time. Simultaneously, we also observed that there was a lagged response between VIs and climate variables. In line with this idea, most studies indicate an (up to) 3 months lagged relationship between VIs response and climate variable effect [67]. The range of the period appears to be related to the studied area’s specific characteristics, such as climate, topography, and soil type, affecting VI lagged response [77,78].

The VIs seasonal behaviour also plays a crucial role in the VIs dynamics [79]; therefore, we obtained different strengths in the relationship between VIs and climate over the year. As Helman et al. [80] reported, NDVI showed a better response to grassland vegetation during wet seasons due to the herbaceous vegetation’s growth in the Mediterranean climate. This effect might explain the higher correlation found in P3 and P5 phases, coincident with the Mediterranean weather’s wet seasons. The same idea might be suggested to the differences between ZMA and ZAV, being ZMA wetter than ZAV.

We achieved better precipitation correlations when the year was divided into phases, although they were not as high as the temperature. This fact is consistent with [81] work suggesting that Mediterranean precipitation is characterised by a complex seasonal variability pattern, with large and unpredictable rainfall fluctuations from one year to the other, hindering the relationship between precipitation and VIs.

Once the year is divided into phases, our results highlight a variable lag’s usefulness depending on the year’s season to characterise the vegetation–climate system. This result agrees with Zhang et al., study [33] that supports the idea of a variable *ℓ* along the time. In their case, *ℓ* varied from 0 to 90 days depending on the season and the climate variable.

In line with this idea, [62] suggested that the season of the year and the type of vegetation cover are critical in the *ℓ* estimation. Even more, other authors indicate that local conditions are crucial in the estimation of *ℓ*. Suzuki et al. [71] revealed that the NDVI lagged response changed inside the same study area. In the northern, NDVI varied because of the warmth variations. On the other hand, in the southern, NDVI varied due to the inter-annual wetness fluctuations instead of warmth changes.

From another point of view, several authors reported that *ℓ* depends on the observed time scale. Cui and Shi [7] found a 30-day NDVI lagged response to precipitation. Meanwhile, Wang et al. [6] suggested that the bi-weekly lag was the most correlated. As we stated before, it is essential to note that these relationships were found in these local conditions and the proposed phases. If the analysis is applied to a broader area, the relationships might not be statistically significant as the conditions could differ in space and time [82].

We already emphasised the incredible complexity of the vegetation–climate system in the previous analysis. Ecological systems present nonlinear dynamics, combining chaotic and periodic cycles, whose equations controlling the systems are unknown [83,84]. Thus, the nonlinear analysis provides complementary information about the system. In our work, we detected a dimensionality increase in MSAVI RPs. More detailed soil information was introduced through L in the MSAVI series; thus, a higher embedding dimension was expected. This result agrees with previous literature findings [85,86], which relate dimensionality increases to complexity increases. In this line, Marwan et al. [87] demonstrated the usefulness of RPs to describe nonlinear behaviours in high-dimensional systems, such as VI time-series.

In MSAVI RPs, we found white stripes in the RPs pattern. These structures are related to atypical values and an interruption in the vegetation pattern [42]. We believe that this behaviour is due to an extreme climatic event that increased soil moisture; consequently, VI series values atypically increased, being detected in the MSAVI RPs.

Diagonal-wise profiles of the CRPs revealed the maximum lag in each zone, and it is expected to vary between zones, seasons, and vegetation cover. Several lags have been reported in the literature. Cao et al. [77] reported a twenty-day lag in precipitation and temperature in Xinjiang’s arid area (China). In contrast, Richard and Poccard [88] reported a maximum lag of three months in South Africa.

Other authors refer to seasons as the most crucial factor in the variation of maximum lag. As reported by Zhao et al. [89], precipitation showed a 1–2 month lag in spring, whereas maximum lag is reduced to 1 month in the Autumn season. They also revealed that temperature showed the same lag as precipitation in the spring; however, maximum lag might be increased up to 3 months in autumn. This result is in concordance with the recurrence profile results, where the temperature did not show an evident maximum lag in the first 50 days.

The CRQA analysis allowed us to quantify the different dynamics of the VIs and climate variables. The DET value has been utilised to indicate climate stability [45] or detecting bioclimatic transitions [40]. The MSAVI time-series showed a higher value of DET in comparison to NDVI. Our results suggest that MSAVI allowed us to characterise the semiarid grasslands better. We speculate that soil moisture is being detected by the MSAVI index, allowing us to improve the NDVI results in ZMA. The same phenomena happened in the VI-PCP CRPs, where MSAVI achieved a higher DET value than NDVI in ZMA. Our results agree with Marwan et al. [87] that found a higher DET in a humid grassland area than a dry grassland.

Regarding LT, both VIs obtained low values compared to the periodic series [40]. This fact might indicate that vegetation may be predicted in the short term due to the incredible complexity of ecological systems, as reported by Beckage et al. [90].

Now, let us discuss the values of ENTR, which refer to the disorder of the system. Standard values obtained by Zhao et al. [40] noted that stochastic systems tend to obtain lower ENTR values (0.2) in comparison with those of periodic systems (2.20). We speculate that the high value of ENTR in the MSAVI case (see Table 8) is the consequence of the high number of precipitations in ZMA. Marwan et al. [87] sustained this fact, suggesting that wet grassland areas tend to obtain higher ENTR values than dry grassland areas.

The LAM and TT are related to the vertical structures created in the RPs and CRPs. LAM refers to the chaos transitions and represents the number of laminar states [91]. MSAVI presents a higher value than NDVI, indicating that values are trapped during specific periods, decreasing time-series dispersion, and supporting the idea of higher predictability and determinism of the MSAVI index. TT represents the average length of vertical structures and indicates how long the state will be trapped, while MSAVI showed a higher TT value than NDVI. We believe that this fact is the consequence of the behaviour of each time series. MSAVI is less dispersed, and then it is expected to be trapped in similar states much longer than NDVI. The same principle might be applied to temperature and precipitation. Temperature is seasonal and did not dramatically change between two consecutive measures. In contrast, precipitation is erratic, and it is not equally distributed over time [81], especially in the Mediterranean climate.

Overall, our results highlight the incredible complexity of the grassland system. We observed that the time scale is a critical component in the analysis of the VIs series. At the same time, we prove that RPs, CRPs and CRQA are a promising analysis that could provide complementary information about the system dynamics that the linear methods could not describe.

## 5. Conclusions

The VIs time series has an exciting potential to assess the grassland’s response to environmental factors, especially to climate variables. The relationships between VIs and the climate variables studied here depend on the years’ time and the climate variable. The vegetation–climate complexity suggests that each area presents its features and local climate conditions. Thus, correlation analysis between VIs and climate variables should not be restricted to year seasons.

We applied the cross-correlation method to characterise the vegetation–climate system’s complexity, concluding that temperature is the most decisive driver factor in this case. However, it is essential to note that precipitation showed a stable positive trend along the phases suggesting that precipitation events are beneficial in arid-semiarid grassland growth, regardless of the years’ time. Even though it is challenging to study due to its irregular temporal distribution, precipitation showed a significant positive correlation in phase 3 at the end of the spring. This correlation increased when MSAVI was used.

The RP, CRPs and RQA were applied to VIs time series to measure the complexity of the dynamics in the grassland–climate system. Both indices showed differences between each zone. However, we detected a characteristic dynamic that points out short-term predictability and high-dimensionality of the MSAVI time-series. Moreover, this analysis allowed us to differentiate the precipitation regime that affected MSAVI dynamics. This fact is shown in the CRPs, pointing out a better grassland characterisation in the wet zone (ZMA) by MSAVI, likely due to the influence of soil water availability.

Overall, the addition of bare soil influence and non-photosynthetic vegetation residuals was beneficial to VIs sensitivity in semiarid pastures. However, further research is needed in different areas and distinct types of pastures.

## Figures and Tables

**Table 1:** Annual climate and soil characteristics of both study zones. ZAV: Tornadizos de Ávila (Avila), ZMA: Soto Del Real (Madrid).

Variable	ZAV	ZMA
Slope (%)	4.2 (±1.10)	4.7 (±1.60)
Height (m)	1290 (±70)	958 (±30)
Silt (%)	20 (±2.00)	18 (±1.00)
Sand (%)	60 (±2.00)	76 (±1.00)
Clay (%)	20 (±2.00)	6 (±1.00)
Bulk Density (g/cm^3^)	1.3 (±0.10)	1.6 (±0.10)
pH	6.5 (±0.30)	5.6 (±0.20)
Organic Matter (%)	3.8 (±0.20)	3.0 (±0.10)
Water Holding Capacity (%)	14.4 (±1.00)	11.1 (±1.00)
Precipitation (mm)	400 (±150.00)	560 (±80.00)
Temperature (°C)	11.6 (±0.60)	13.6 (±0.60)

**Table 2:** Annual pasture phases based on vegetation index (VIs) trend at the studied zones (Tornadizos de Ávila and Soto del Real).

Code Phase	Initial Date	Final Date	Months Implied	Seasons Implied	VIs Trend
P1	25 November	25 January	November-December-January	Autumn–Winter	Constant
P2	2 February	23 April	February-March-April	Winter–Spring	Increasing
P3	1 May	20 June	May-June	Spring–Summer	Decreasing
P4	28 July	22 September	July-August-September	Summer–Autumn	Constant
P5	30 September	17 November	September-October-November	Autumn	Increasing

**Table 3:** Time-series Pearson correlation coefficients (CR) and partial correlation coefficients (PCR) between Modified Soil-Adjusted Vegetation Index (MSAVI) and meteorological time-series for each study zone (ZAV: Tornadizos de Ávila; ZMA: Soto del Real) during the period of 2002–2018. TEMP is 8-day average air temperature (°C), and PCP is the accumulated precipitation in 8-day (mm).

Zone	CR		PCR	
	TEMP	PCP	TEMP	PCP
ZAV	−0.435 **	0.165 **	−0.571 **	0.199 **
ZMA	−0.572 **	0.199 **	−0.504 **	0.074 **

Note: ** represents *p* < 0.01 significance.

**Table 4:** Pearson correlation coefficients (CR) and partial correlation coefficients (PCR) of MSAVI and meteorological parameters in five distinct phases. TEMP is 8-day average air temperature (°C), and PCP is the accumulated precipitation in 8-day (mm). ZAV is Tornadizos de Ávila, and ZMA is Soto del Real.

		CR		PCR	
	PHASE	MSAVI xTEMP	MSAVI x PCP	MSAVI x TEMP	MSAVI x PCP
ZAV	P1	0.130	0.046	0.173*	0.008
	P2	0.489 **	0.066	−0.027	0.180 *
	P3	−0.714 **	0.283 **	−0.095	−0.003
	P4	0.270 **	−0.037	−0.130	0.106
	P5	−0.550 **	0.216 *	0.072	−0.052
ZMA	P1	0.101	0.023	0.144	0.015
	P2	0.428 **	0.042	0.026	0.125
	P3	−0.763 **	0.359 **	−0.007	−0.053
	P4	0.248 **	0.029	0.007	0.135
	P5	−0.594 **	0.160	0.040	−0.157

Note: * represents *p* < 0.05 significance, ** represents *p* < 0.01 significance.

**Table 5:** Cross-correlation coefficients between MSAVI and meteorological parameters with different lags (*ℓ*) in different phases. ZAV: Tornadizos de Ávila and ZMA: Soto del Real. TEMP is 8-day average air temperature (°C), and PCP is the accumulated precipitation in 8-day (mm). The bold number represents the maximum correlation in each row. Each time lag is of 8 days.

			Time Lag (*ℓ*)						
	Phase	Param.	0	1	2	3	4	5	6
ZAV	P2	TEMP	**0.492**	0.451	0.418	0.333	0.271	0.198	0.041
		PCP	0.067	0.039	0.074	**0.133**	−0.031	−0.003	0.079
	P3	TEMP	−0.714	−0.739	**−0.741**	−0.726	−0.714	−0.681	−0.629
		PCP	0.283	0.312	**0.378**	0.243	0.256	0.186	0.064
	P5	TEMP	−0.550	−0.553	−0.575	−0.584	−0.605	**−0.613**	−0.561
		PCP	0.216	0.359	0.396	**0.424**	0.235	0.214	0.241
ZMA	P2	TEMP	**0.422**	0.308	0.259	0.233	0.169	0.109	0.029
		PCP	0.032	0.088	0.186	**0.221**	0.156	0.102	0.093
	P3	TEMP	**−0.763**	−0.757	−0.717	−0.699	−0.695	−0.697	−0.637
		PCP	0.359	**0.396**	0.381	0.321	0.357	0.309	0.230
	P5	TEMP	−0.594	−0.600	**−0.625**	−0.602	−0.603	−0.581	−0.512
		PCP	0.159	0.297	0.393	**0.397**	0.293	0.275	0.304

**Table 6:** Recurrence plot (RP) parameters in ZAV: Tornadizos de Ávila and ZMA: Soto del Real. MSAVI: Modified Soil-Adjusted Vegetation Index, *m*: Embedding dimension, τ: Delay, r: threshold.

Zone	RPs	*m*	τ	r	RR (%)
ZAV	MSAVI	6	9	20.91	4.99
	TEMP	2	11	8.76	4.99
	PCP	2	3	1.35	4.97
ZMA	MSAVI	8	11	26.32	4.99
	TEMP	2	11	9.00	4.99
	PCP	10	9	13.25	5.00

**Table 7:** Cross-Recurrence plot (RP) parameters in ZAV: Tornadizos de Ávila and ZMA: Soto del Real. MSAVI-TEMP: Cross-Recurrence plot between MSAVI and Average temperature. MSAVI-PCP: Cross-Recurrence plot between MSAVI and Accumulated precipitation.

Zone	CRPs	*m*	τ	r	RR (%)
ZAV	MSAVI-TEMP	6	11	23.04	5.00
	MSAVI-PCP	6	11	17.27	5.00
ZMA	MSAVI-TEMP	8	11	25.60	5.00
	MSAVI-PCP	10	11	23.74	5.00

**Table 8:** Recurrence plot (RP) and Cross Recurrence plots (CRP) parameters and Recurrence Quantification Analysis (RQA) using general z-score vegetation indices series in ZAV: Tornadizos de Ávila and ZMA: Soto del Real. MSAVI: Modified Soil-Adjusted Vegetation Index, m: Embedding dimension, τ: Delay, r: threshold, RR: Recurrence rate, DET: Determinism, LT: Average length of diagonal structures, ENTR: Shannon Entropy, LAM: Laminarity, TT: Trapping time.

Zone	RPs and CRPs	RR (%)	DET (%)	LT	ENTR	LAM (%)	TT
ZAV	MSAVI	4.99	73.20	3.40	1.57	82.66	3.55
	TEMP	4.99	40.26	2.31	0.72	52.89	2.49
	PCP	4.97	14.65	2.10	0.32	33.72	2.35
	MSAVI-TEMP	5.00	63.75	2.88	1.29	75.30	3.62
	MSAVI-PCP	5.00	19.25	2.17	0.47	23.08	2.27
ZMA	MSAVI	4.99	78.42	4.17	1.80	86.13	3.88
	TEMP	4.99	42.02	2.35	0.76	54.35	2.60
	PCP	5.00	6.57	2.03	0.14	20.72	2.11
	MSAVI-TEMP	5.00	69.06	3.21	1.50	76.59	3.75
	MSAVI-PCP	5.00	25.70	2.26	0.64	29.23	2.29

## Data Availability

The data presented in this study are available on request from the corresponding author.

## Supplementary Materials

The following are available online at https://www.mdpi.com/article/10.3390/e23050559/s1, Figure S1: title, Table S1: Statistical trends significance of vegetation indices (NDVI and MSAVI) and climatic variables, Table S2. Chow’s test of annual pasture phases, Figure S1. Growing phases response of NDVI to water (Accumulated precipitation) and energy (Average temperature), Table S3. Time-series Pearson correlation coefficients (CR) and partial correlation coefficients (PCR) between NDVI time-series and meteorological time-series, Figure S2. Time series cross-correlation between NDVI and climatic variables time series (Temperature and Accumulated precipitation), Table S4. Pearson correlation coefficients (CR) and partial correlation coefficients (PCR) between NDVI and meteorological parameters in five distinct phases, Table S5. Cross-correlation coefficients between NDVI and meteorological parameters with different lags (*ℓ*) in distinct phases, Table S6. Recurrence plot (RP) and Cross Recurrence plots (CRP) parameters and Recurrence Quantification Analysis (RQA) using general z-score vegetation indices series, Figure S3. Optimized recurrence plots (RP) using normalized vegetation indices (NDVI), Average temperature (TEMP) and Accumulated precipitation (PCP) data, Figure S4. Diagonal-wise recurrence profile of the RPs obtained from the NDVI, Average temperature (TEMP) and accumulated precipitation (PCP), Figure S5. Optimized Cross-Recurrence Plots (CRPs) and diagonal-wise recurrence profiles between vegetation indices data (NDVI) temperature data (TEMP) and accumulated precipitation data (PCP).

## Appendix A

In this work, RQA formulas were obtained from Marwan et al. [43]; for further details, it is recommended to revise the work mentioned above. The first step to obtaining RQA measures is the calculation of RP histogram (*P*) from the total number of diagonal lines, of length (*l*), and the total number of vertical lines of length (*v*). The following formulas define these histograms:

(A1) $$P\left(l\right)=\sum_{i,j=1}^{N}\left(1-R_{i-1,j-1}\left(\varepsilon \right)\right)\left(1-R_{i+1,j+1}\left(\varepsilon \right)\right)\prod_{k=0}^{l-1}R_{i+k,j+k}\left(\varepsilon \right),$$

(A2) $$P\left(v\right)=\sum_{i,j=1}^{N}\left(1-R_{i,j}\right)\left(1-R_{i,j+v}\right)\prod_{k=0}^{v-1}R_{i,j+k}$$

DET is the proportion of recurrent points assembling diagonal structures of all recurrence points. It represents the system’s predictability (100% to purely deterministic systems and 0% to random stochastic series). DET is calculated by:

(A3) $$\mathrm{DET}=\frac{\sum_{l=l_{min}}^{N}lP\left(l\right)}{\sum_{i,j}^{N}R_{i,j}},$$

Where *l_min_* is the minimal length to consider a diagonal line, in this case, as suggested by (Webber and Zbilut, 1994), it was established as 2.

LT is the average time passed when two trajectories are close to each other, and it is calculated by the following Equation (4):

(A4) $$\mathrm{LT}=\frac{\sum_{l=l_{min}}^{N}lP\left(l\right)}{\sum_{l=l_{min}}^{N}P\left(l\right)},$$

ENT points out the probability to find the same trajectory of length *l* in the whole RP. The consequent formula is applied (5):

(A5) $$\mathrm{ENTR}=-\sum_{l=l_{min}}^{N}p\left(l\right)lnp\left(l\right)$$

Analogous to the definition of determinism (DET) in Equation (3), LAM represents the number of vertical structures forming the vertical structures. It represents an overall measure of the signal stability, and the following equation calculates it:

(A6) $$\mathrm{LAM}=\frac{\sum_{v=v_{min}}^{N}vP\left(v\right)}{\sum_{v=1}^{N}vP\left(v\right)},$$

where *v_min_* is the minimal length to count a vertical line, in this case, as proposed by Marwan et al. [43], it was set as 2.

The following formula computes the average length of vertical structures (TT):

(A7) $$\mathrm{TT}=\frac{\sum_{v=vmin}^{N}v\;P\left(v\right)}{\sum_{v=vmin}^{N}P\left(v\right)}$$

## References

[1] Konings A.G., Williams A.P., Gentine P. (2017). Sensitivity of grassland productivity to aridity controlled by stomatal and xylem regulation. Nat. Geosci..

[2] Scheuring I., Riedi R.H. (1994). Application of multifractals to the analysis of vegetation pattern. J. Veg. Sci..

[3] Hobbs R.J., Yates S., Mooney H.A. (2007). Long-term data reveal complex dynamics in grassland in relation to climate and disturbance. Ecol. Monogr..

[4] Serrano J., Shahidian S., da Silva J.M. (2019). Evaluation of normalised difference water index as a tool for monitoring pasture seasonal and inter-annual variability in a Mediterranean agro-silvo-pastoral system. Water.

[5] Li B., Tao S., Dawson R.W. (2002). Relations between AVHRR NDVI and ecoclimatic parameters in China. Int. J. Remote Sens..

[6] Wang J., Rich P.M., Price K.P. (2003). Temporal responses of NDVI to precipitation and temperature in the central Great Plains, USA. Int. J. Remote Sens..

[7] Cui L., Shi J. (2010). Temporal and spatial response of vegetation NDVI to temperature and precipitation in eastern China. J. Geogr. Sci..

[8] Gessner U., Naeimi V., Klein I., Kuenzer C., Klein D., Dech S. (2013). The relationship between precipitation anomalies and satellite-derived vegetation activity in Central Asia. Glob. Planet. Chang..

[9] Braswell B.H., Schimel D.S., Linder E., Moore B. (1997). The Response of Global Terrestrial Ecosystems to Interannual Temperature Variability. Science.

[10] Piao S., Mohammat A., Fang J., Cai Q., Feng J. (2006). NDVI-based increase in growth of temperate grasslands and its responses to climate changes in China. Glob. Environ. Chang..

[11] Liu Y., Zha Y., Gao J., Ni S. (2004). Assessment of grassland degradation near Lake Qinghai, West China, using Landsat TM and in situ reflectance spectra data. Int. J. Remote Sens..

[12] Hively W.D., Lamb B.T., Daughtry C.S.T., Shermeyer J., McCarty G.W., Quemada M. (2018). Mapping crop residue and tillage intensity using WorldView-3 satellite shortwave infrared residue indices. Remote Sens..

[13] Blanco L.J., Paruelo J.M., Oesterheld M., Biurrun F.N. (2016). Spatial and temporal patterns of herbaceous primary production in semiarid shrublands: A remote sensing approach. J. Veg. Sci..

[14] Steele-Dunne S.C., McNairn H., Monsivais-Huertero A., Judge J., Liu P.W., Papathanassiou K. (2017). Radar Remote Sensing of Agricultural Canopies: A Review. IEEE J. Sel. Top. Appl. Earth Obs. Remote Sens..

[15] Liu S., Gao L., Lei Y., Wang M., Hu Q., Ma X., Zhang Y. (2020). SAR Speckle Removal Using Hybrid Frequency Modulations. IEEE Trans. Geosci. Remote Sens..

[16] Das A., Agrawal R., Mohan S. (2015). Topographic correction of ALOS-PALSAR images using InSAR-derived DEM. Geocarto Int..

[17] Mao D., Wang Z., Luo L., Ren C. (2012). Integrating AVHRR and MODIS data to monitor NDVI changes and their relationships with climatic parameters in Northeast China. Int. J. Appl. Earth Obs. Geoinf..

[18] Guo B., Zhou Y., Wang S., Tao H. (2014). The relationship between normalised difference vegetation index (NDVI) and climate factors in the semiarid region: A case study in Yalu Tsangpo River basin of Qinghai-Tibet Plateau. J. Mt. Sci..

[19] Rouse J.W., Haas R.H., Schell J.A., Deering D.W. (1973). Monitoring the vernal advancement and retrogradation (green wave effect) of natural vegetation. Progress Report RSC 1978-1. https://core.ac.uk/download/pdf/80640125.pdf.

[20] Huete A., Didan K., Miura T., Rodriguez E.P., Gao X., Ferreira L.G. (2002). Overview of the radiometric and biophysical performance of the MODIS vegetation indices. Remote Sens. Environ..

[21] Yang L., Wylie B.K., Tieszen L.L., Reed B.C. (1998). An analysis of relationships among climate forcing and time-integrated NDVI of grasslands over the U.S. northern and central Great Plains. Remote Sens. Environ..

[22] Jafari R., Bashari H., Tarkesh M. (2017). Discriminating and monitoring rangeland condition classes with MODIS NDVI and EVI indices in Iranian arid and semiarid lands. Arid Land Res. Manag..

[23] Yagci A.L., Di L., Deng M. The influence of land cover-related changes on the NDVI-based satellite agricultural drought indices. Proceedings of the 2014 IEEE Geoscience and Remote Sensing Symposium.

[24] Fern R.R., Foxley E.A., Bruno A., Morrison M.L. (2018). Suitability of NDVI and OSAVI as estimators of green biomass and coverage in a semiarid rangeland. Ecol. Indic..

[25] Huete A.R., Jackson R.D., Post D.F. (1985). Spectral response of a plant canopy with different soil backgrounds. Remote Sens. Environ..

[26] Eisfelder C., Kuenzer C., Dech S. (2012). Derivation of biomass information for semi-arid areas using remote-sensing data. Int. J. Remote Sens..

[27] Ren H., Zhou G., Zhang F. (2018). Using negative soil adjustment factor in soil-adjusted vegetation index (SAVI) for above-ground living biomass estimation in arid grasslands. Remote Sens. Environ..

[28] Qi J., Chehbouni A., Huete A.R., Kerr Y.H., Sorooshian S. (1994). A modified soil adjusted vegetation index. Remote Sens. Environ..

[29] Jiang Y., Tao J., Huang Y., Zhu J., Tian L., Zhang Y. (2013). The spatial pattern of grassland above-ground biomass on Xizang Plateau and its climatic controls. J. Plant. Ecol..

[30] Li G., Wang J., Wang Y., Wei H., Ochir A., Davaasuren D., Chonokhuu S., Nasanbat E. (2019). Spatial and temporal variations in grassland production from 2006 to 2015 in Mongolia along the China-Mongolia Railway. Sustainability.

[31] Chamaille-Jammes S., Fritz H., Murindagomo F. (2006). Spatial patterns of the NDVI-rainfall relationship at the seasonal and interannual time scales in an African savanna. Int. J. Remote Sens..

[32] Ma X., Huete A., Yu Q., Coupe N.R., Davies K., Broich M., Ratana P., Beringer J., Hutley L.B., Cleverly J. (2013). Spatial patterns and temporal dynamics in savanna vegetation phenology across the north australian tropical transect. Remote Sens. Environ..

[33] Zhang Y., Wang X., Li C., Cai Y., Yang Z., Yi Y. (2018). NDVI dynamics under changing meteorological factors in a shallow lake in future metropolitan, semiarid area in North China. Sci. Rep..

[34] Chen Z., Wang W., Fu J. (2020). Vegetation response to precipitation anomalies under different climatic and biogeographical conditions in China. Sci. Rep..

[35] Cong N., Shen M., Yang W., Yang Z., Zhang G., Piao S. (2017). Varying responses of vegetation activity to climate changes on the Tibetan Plateau grassland. Int. J. Biometeorol..

[36] Wang H., Liu D., Lin H., Montenegro A., Zhu X. (2015). NDVI and vegetation phenology dynamics under the influence of sunshine duration on the Tibetan plateau. Int. J. Climatol..

[37] Shen B., Fang S., Li G. (2014). Vegetation coverage changes and their response to meteorological variables from 2000 to 2009 in Naqu, Tibet, China. Can. J. Remote Sens..

[38] Martínez B., Gilabert M.A. (2009). Vegetation dynamics from NDVI time series analysis using the wavelet transform. Remote Sens. Environ..

[39] Zhong L., Ma Y., Salama M.S., Su Z. (2010). Assessment of vegetation dynamics and their response to variations in precipitation and temperature in the Tibetan Plateau. Clim. Chang..

[40] Zhao Z., Liu J., Peng J., Li S., Wang Y. (2015). Nonlinear features and complexity patterns of vegetation dynamics in the transition zone of North China. Ecol. Indic..

[41] Eckmann J.P., Oliffson Kamphorst O., Ruelle D. (1987). Recurrence plots of dynamical systems. Europhys. Lett..

[42] Proulx R., Parrott L., Fahrig L., Currie D.J., Webber C.L., Marwan N. (2015). Long Time-Scale Recurrences in Ecology: Detecting Relationships Between Climate Dynamics and Biodiversity Along a Latitudinal Gradient. Recurrence Quantification Analysis—Theory and Best Practices.

[43] Marwan N., Carmen Romano M., Thiel M., Kurths J. (2007). Recurrence plots for the analysis of complex systems. Phys. Rep..

[44] Proulx R., Cöté P., Parrott L. (2008). Use of recurrence analysis to measure the dynamical stability of a multi-species community model. Eur. Phys. J. Spec. Top..

[45] Li S.C., Zhao Z.Q., Liu F.Y. (2008). Identifying spatial pattern of NDVI series dynamics using recurrence quantification analysis. Eur. Phys. J. Spec. Top..

[46] Zurlini G., Marwan N., Semeraro T., Jones K.B., Aretano R., Pasimeni M.R., Valente D., Mulder C., Petrosillo I. (2018). Investigating landscape phase transitions in Mediterranean rangelands by recurrence analysis. Landsc. Ecol..

[47] Semeraro T., Luvisi A., Lillo A.O., Aretano R., Buccolieri R., Marwan N. (2020). Recurrence analysis of vegetation indices for highlighting the ecosystem response to drought events: An application to the amazon forest. Remote Sens..

[48] LP DAAC (2014). Land Processes Distributed Active Archive Center: Surface Reflectance 8-Day L3 Global 500 m, NASA and USGS. https://lpdaac.usgs.gov/products/mod09a1v006/.

[49] Martín-Sotoca J.J., Saa-Requejo A., Moratiel R., Dalezios N., Faraslis I., Tarquis A.M. (2019). Statistical analysis for satellite-index-based insurance to define damaged pasture thresholds. Nat. Hazards Earth Syst. Sci..

[50] Xu D., Guo X. (2013). A study of soil line simulation from landsat images in mixed grassland. Remote Sens..

[51] Xu M., Eckstein Y. (1995). Use of Weighted Least-Squares Method in Evaluation of the Relationship Between Dispersivity and Field Scale. Groundwater.

[52] Agencia Estatal de Meteorología AEMET OpenData. https://opendata.aemet.es/centrodedescargas/inicio.

[53] Tarquis A.M., Castellanos M.T., Cartagena M.C., Arce A., Ribas F., Cabello M.J., De Herrera J.L., Bird N.R.A. (2017). Scale and space dependencies of soil nitrogen variability. Nonlinear Process. Geophys..

[54] Chow G.C. (1960). Tests of Equality Between Sets of Coefficients in Two Linear Regressions. Econom. J. Econom. Soc..

[55] Coco M.I., Dale R. (2014). Cross-recurrence quantification analysis of categorical and continuous time series: An R package. Front. Psychol..

[56] Marwan N. CRP Toolbox 5.22 (R32.4). http://tocsy.pik-potsdam.de/CRPtoolbox/.

[57] Webber C.L., Zbilut J. (2005). Recurrence quantification analysis of nonlinear dynamical systems. Tutorials in Contemporary Nonlinear Methods for the Behavioral Sciences Web Book.

[58] Fraser A.M., Swinney H.L. (1986). Independent coordinates for strange attractors from mutual information. Phys. Rev. A.

[59] Cao L. (1997). Practical method for determining the minimum embedding dimension of a scalar time series. Phys. D Nonlinear Phenom..

[60] Baret F., Jacquemoud S., Hanocq J.F. (1993). About The soil line concept in remote sensing. Adv. Sp. Res..

[61] Quemada M., Hively W.D., Daughtry C.S.T., Lamb B.T., Shermeyer J. (2018). Improved crop residue cover estimates obtained by coupling spectral indices for residue and moisture. Remote Sens. Environ..

[62] Chuai X.W., Huang X.J., Wang W.J., Bao G. (2013). NDVI, temperature and precipitation changes and their relationships with different vegetation types during 1998–2007 in Inner Mongolia, China. Int. J. Climatol..

[63] Hao F., Zhang X., Ouyang W., Skidmore A.K., Toxopeus A.G. (2012). Vegetation NDVI Linked to Temperature and Precipitation in the Upper Catchments of Yellow River. Environ. Model. Assess..

[64] Vicente-Serrano S.M., Tomas-Burguera M., Beguería S., Reig F., Latorre B., Peña-Gallardo M., Luna M.Y., Morata A., González-Hidalgo J.C. (2017). A high resolution dataset of drought indices for Spain. Data.

[65] Estrela T., Vargas E. (2012). Drought Management Plans in the European Union. The Case of Spain. Water Resour. Manag..

[66] Pang G., Wang X., Yang M. (2017). Using the NDVI to identify variations in, and responses of, vegetation to climate change on the Tibetan Plateau from 1982 to 2012. Quat. Int..

[67] Xu Y., Yang J., Chen Y. (2016). NDVI-based vegetation responses to climate change in an arid area of China. Theor. Appl. Climatol..

[68] Liu Y., Li Y., Li S., Motesharrei S. (2015). Spatial and temporal patterns of global NDVI trends: Correlations with climate and human factors. Remote Sens..

[69] Huete A.R., Asrar G. (1989). Soil influences in remotely sensed vegetation-canopy spectra. Theory and Applications of Optical Remote Sensing.

[70] Sun J., Qin X. (2016). Precipitation and temperature regulate the seasonal changes of NDVI across the Tibetan Plateau. Environ. Earth Sci..

[71] Suzuki R., Masuda K.G., Dye D. (2007). Interannual covariability between actual evapotranspiration and PAL and GIMMS NDVIs of northern Asia. Remote Sens. Environ..

[72] Viola F., Daly E., Vico G., Cannarozzo M., Porporato A. (2008). Transient soil-moisture dynamics and climate change in Mediterranean ecosystems. Water Resour. Res..

[73] Grant K., Kreyling J., Dienstbach L.F.H., Beierkuhnlein C., Jentsch A. (2014). Water stress due to increased intra-annual precipitation variability reduced forage yield but raised forage quality of a temperate grassland. Agric. Ecosyst. Environ..

[74] Fu B., Burgher I. (2015). Riparian vegetation NDVI dynamics and its relationship with climate, surface water and groundwater. J. Arid Environ..

[75] Sala O.E., Parton W.J., Joyce L.A., Lauenroth W.K. (1988). Primary Production of the Central Grassland Region of the United States. Ecology.

[76] Olmos-Trujillo E., González-Trinidad J., Júnez-Ferreira H., Pacheco-Guerrero A., Bautista-Capetillo C., Avila-Sandoval C., Galván-Tejada E. (2020). Spatio-temporal response of vegetation indices to rainfall and temperature in a semiarid region. Sustainbility.

[77] Cao X.M., Chen X., Bao A.M., Wang Q. (2011). Response of vegetation to temperature and precipitation in Xinjiang during the period of 1998–2009. J. Arid Land.

[78] Guo L., Wu S., Zhao D., Yin Y., Leng G., Zhang Q. (2014). NDVI-Based Vegetation Change in Inner Mongolia from 1982 to 2006 and Its Relationship to Climate at the Biome Scale. Adv. Meteorol..

[79] Gong Z., Kawamura K., Ishikawa N., Goto M., Wulan T., Alateng D., Yin T., Ito Y. (2015). MODIS normalised difference vegetation index (NDVI) and vegetation phenology dynamics in the Inner Mongolia grassland. Solid Earth.

[80] Helman D., Lensky I.M., Tessler N., Osem Y. (2015). A phenology-based method for monitoring woody and herbaceous vegetation in mediterranean forests from NDVI time series. Remote Sens..

[81] Ramos M.C. (2001). Rainfall distribution patterns and their change over time in a Mediterranean area. Theor. Appl. Climatol..

[82] Meroni M., Rembold F., Verstraete M.M., Gommes R., Schucknecht A., Beye G. (2014). Investigating the relationship between the inter-annual variability of satellite-derived vegetation phenology and a proxy of biomass production in the Sahel. Remote Sens..

[83] Proulx R., Parrott L. (2009). Structural complexity in digital images as an ecological indicator for monitoring forest dynamics across scale, space and time. Ecol. Indic..

[84] Storch D., Gaston K.J. (2004). Untangling ecological complexity on different scales of space and time. Basic Appl. Ecol..

[85] Belaire-Franch J., Contreras D., Tordera-Lledó L. (2002). Assessing nonlinear structures in real exchange rates using recurrence plot strategies. Phys. D Nonlinear Phenom..

[86] Donner R.V., Zou Y., Donges J.F., Marwan N., Kurths J. (2010). Recurrence networks—A novel paradigm for nonlinear time series analysis. New J. Phys..

[87] Marwan N., Kurths J., Foerster S. (2015). Analysing spatially extended high-dimensional dynamics by recurrence plots. Phys. Lett. A.

[88] Richard Y., Poccard I. (1998). A statistical study of NDVI sensitivity to seasonal and interannual rainfall variations in Southern Africa. Int. J. Remote Sens..

[89] Zhao J., Huang S., Huang Q., Wang H., Leng G., Fang W. (2020). Time-lagged response of vegetation dynamics to climatic and teleconnection factors. Catena.

[90] Beckage B., Gross L.J., Kauffman S. (2011). The limits to prediction in ecological systems. Ecosphere.

[91] Marwan N., Wessel N., Meyerfeldt U., Schirdewan A., Kurths J. (2002). Recurrence-plot-based measures of complexity and their application to heart-rate-variability data. Phys. Rev. E.

