# Supplementary material for: The Vegetation–Climate System Complexity through Recurrence Analysis
Source: Entropy (Basel). 2021 Apr 30;23(5):559. doi: 10.3390/e23050559 (PMC8145696; doi:10.3390/e23050559)
Supplement: Supplementary file 1 [file entropy-23-00559-s001.zip › entropy-1184364-supplementary.pdf]

Table S1. Statistical trends significance of vegetation indices ((Normalised Difference Vegetation Index—NDVI, modified soil-adjusted vegetation index—MSAVI)) and climatic variables (TEMP: Temperature; PCP: Precipitation) during the study period in ZAV: Tornadizos de Ávila and ZMA: Soto del Real.

Zone	Variable	Slope	Standard Error	R^2	t-estimated	t-value	Signif*
ZAV	NDVI	-0.0014	0.0013	0.0678	1.0446	2.1314	n.s.
	MSAVI	-0.001	0.0011	0.0573	0.9552	2.1314	n.s.
	TEMP	0.0384	0.0262	0.1254	1.4665	2.1314	n.s.
	PCP	-2.613	4.2582	0.0245	0.6137	2.1314	n.s.
ZMA	NDVI	0.0001	0.0019	0.0002	0.0529	2.1314	n.s.
	MSAVI	-0.0003	0.0015	0.0032	0.2186	2.1314	n.s.
	TEMP	0.0563	0.0277	0.2158	2.0318	2.1314	n.s.
	PCP	-5.7312	7.7954	0.0348	0.7352	2.1314	n.s.

Note: *Significance at 95% level of confidence: s, significant; n.s., no significant

Table S2. Chow's test of annual pasture phases. ZAV: Tornadizos de Ávila, ZMA: Soto Del Real. REG_1 (linear regression of the first phase data), REG_2 (linear regression of the second phase data, REG_T (both data regression.) B_0 is the intercept, and B_1 is the slope of the linear regression.

			**P1-P2**	**P2-P3**	**P3-P4**	**P4-P5**	**P5-P1**
ZAV	REG_1	B0	0.507**	0.416**	1.088**	0.372**	-0.861**
		B1	-0.003*	0.013**	-0.029**	-0.002*	0.034**
	REG_2	B0	0.414**	1.088**	0.372**	-0.832**	0.507**
		B1	0.013**	-0.029**	-0.002*	0.033**	-0.003*
	REG_T	B0	0.502**	0.606**	0.906**	-0.158**	0.047 *
		B1	0.004**	-0.006**	-0.020**	0.015**	0.010**
	T-Chow	F-chow	25.178*	197.0213*	51.74677*	56.88499*	38.264*
		F-test	3.024	3.020119	3.024042	3.031773	3.032
ZMA	REG_1	B0	0.580**	0.523**	1.298**	0.302**	-1.389**
		B1	0.001*	0.010**	-0.040**	-0.001*	0.049**
	REG_2	B0	0.524**	1.298**	0.302**	-1.393**	0.580**
		B1	0.009**	-0.040**	-0.001*	0.049**	0.001*
	REG_T	B0	0.576**	0.762**	0.947**	-0.451**	-0.085*
		B1	0.004**	-0.014**	-0.022**	0.024**	0.014**
	T-Chow	F-chow	6.189*	174.1243*	83.0957*	60.80033*	40.837**
		F-test	3.024	3.020119	3.024042	3.031773	3.032

Note: *represents P < 0.05 significance, **represents P < 0.01 significance.

(a)

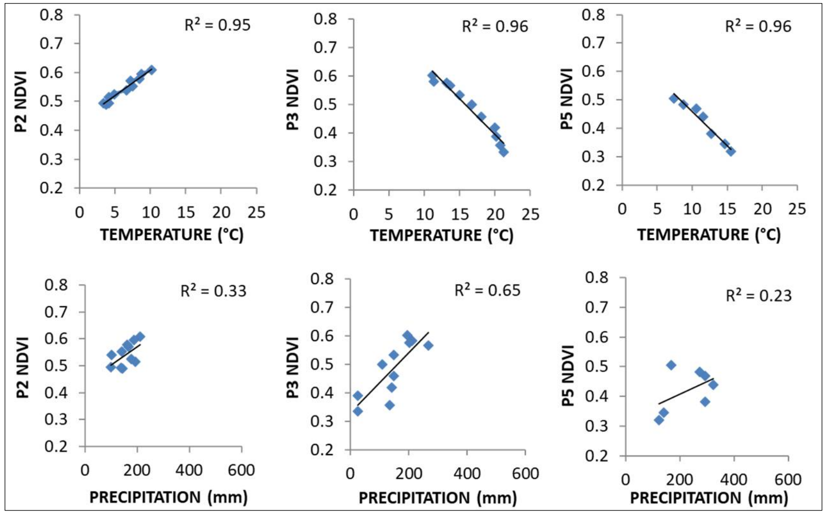

P2 NDVI
P3 NDVI
P5 NDVI
TEMPERATURE (°C)
PRECIPITATION (mm)
R² = 0.95
R² = 0.96
R² = 0.96
R² = 0.33
R² = 0.65
R² = 0.23

(b)

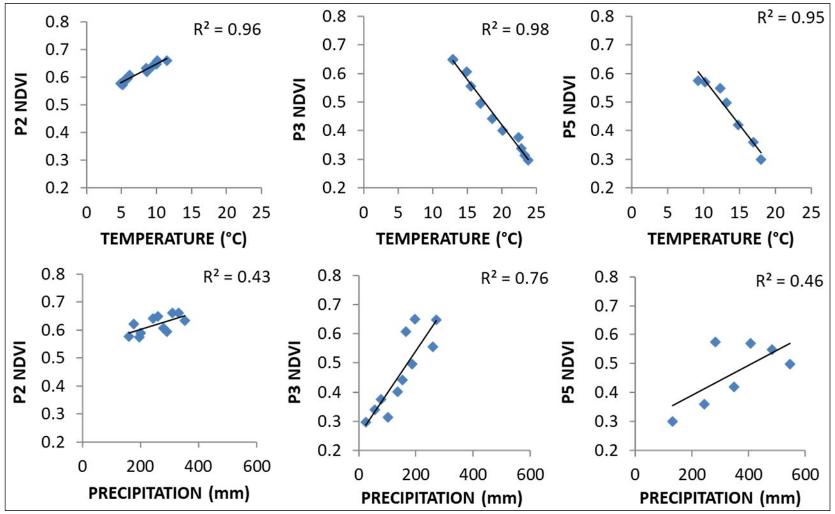

P2 NDVI
P3 NDVI
P5 NDVI
TEMPERATURE (°C)
PRECIPITATION (mm)
R² = 0.96
R² = 0.98
R² = 0.95
R² = 0.43
R² = 0.76
R² = 0.46

Figure S1. Growing phases response of NDVI to water (Accumulated precipitation) and energy (Average temperature) parameters at 8 days in Tornadizos de Ávila (ZAV) **(a)** and Soto del Real (ZMA) **(b)**. In each figure, the regression equation was obtained from the Least Squares method.

Table S3. Time-series Pearson correlation coefficients (CR) and partial correlation coefficients (PCR) between NDVI time-series and meteorological time-series for each study zone (ZAV: Tornadizos de Ávila; ZMA: Soto del Real) during the period of 2002-2018. TEMP is 8-day average air temperature (°C), and PCP is the accumulated precipitation in 8-day (mm).

**Zone**	**CR**		**PCR**	
	TEMP	PCP	TEMP	PCP
ZAV	-0.587**	0.192**	-0.487**	0.168**
ZMA	-0.743**	0.270**	-0.523**	0.104**

Note: **represents P < 0.01 significance

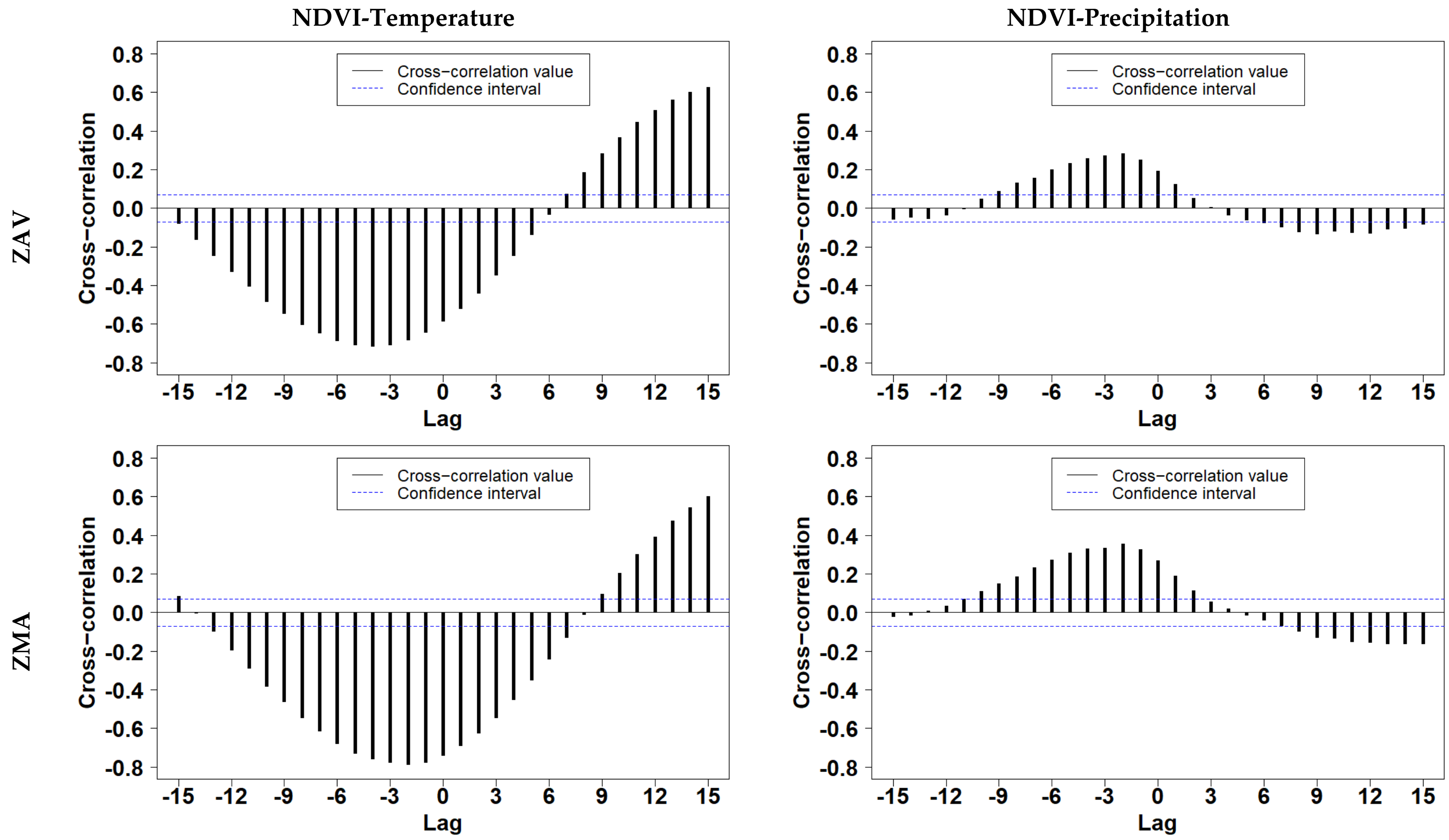

Figure S2. Time series cross-correlation between NDVI and climatic variables time series (Temperature and Accumulated precipitation) in Tornadizos de Ávila (ZAV) and Soto del Real (ZMA). Each lag is of an 8-days period. The blue dot line is the confidence interval at 95%.

Table S4. Pearson correlation coefficients (CR) and partial correlation coefficients (PCR) between NDVI and meteorological parameters in five distinct phases. TEMP is 8-day average air temperature (°C), and PCP is the accumulated precipitation in 8-day (mm). ZAV is Tornadizos de Ávila, and ZMA is Soto del Real.

		**CR**		**PCR**	
	**PHASE**	**NDVI xTEMP**	**NDVI x PCP**	**NDVI x TEMP**	**NDVI x PCP**
ZAV	P1	0.203*	0.029	0.238**	-0.014
	P2	0.524**	0.050	0.082	0.157*
	P3	-0.740**	0.297**	-0.066	-0.010
	P4	0.081	0.109	-0.039	0.150
	P5	-0.578**	0.265**	0.092	-0.010
ZMA	P1	0.040	0.130	0.133	0.105
	P2	0.353**	0.099	0.134	0.180*
	P3	-0.786**	0.402**	0.044	-0.009
	P4	-0.019	0.194*	0.019	0.194*
	P5	-0.654**	0.224*	0.077	-0.126

Note: *represents P < 0.05 significance, **represents P < 0.01 significance.

Table S5. Cross-correlation coefficients between NDVI and meteorological parameters with different lags (ℓ) in different phases. ZAV: Tornadizos de Ávila and ZMA: Soto del Real. TEMP is 8-day average air temperature (°C), and PCP is the accumulated precipitation in 8-day (mm). Each time lag is of 8 days. The bold letter represents the maximum correlation in each row.

			**time lag (ℓ)**						
	**Phase**	**Param.**	**0**	**1**	**2**	**3**	**4**	**5**	**6**
ZAV	P2	TEMP	**0.526**	0.483	0.456	0.314	0.239	0.161	0.026
		PCP	0.049	0.051	**0.116**	0.108	0.053	0.052	0.078
	P3	TEMP	-0.740	-0.762	**-0.773**	-0.756	-0.730	-0.700	-0.659
		PCP	0.297	0.347	**0.401**	0.274	0.269	0.186	0.067
	P5	TEMP	-0.578	-0.592	-0.607	-0.616	-0.632	**-0.635**	-0.567
		PCP	0.265	0.397	**0.451**	0.415	0.249	0.238	0.201
ZMA	P2	TEMP	**0.345**	0.217	0.192	0.174	0.133	0.064	-0.019
		PCP	0.096	0.171	**0.204**	0.198	0.165	0.182	0.148
	P3	TEMP	**-0.786**	-0.784	-0.753	-0.729	-0.715	-0.709	-0.647
		PCP	0.402	0.423	**0.433**	0.369	0.375	0.329	0.225
	P5	TEMP	**-0.654**	-0.650	-0.650	-0.620	-0.645	-0.618	-0.523
		PCP	0.224	0.336	**0.437**	0.375	0.304	0.270	0.249

Table S6. Recurrence plot (RP) and Cross Recurrence plots (CRP) parameters and Recurrence Quantification Analysis (RQA) using general z-score vegetation indices series in ZAV: Tornadizos de Ávila and ZMA: Soto del Real. NDVI: Normalized Difference Vegetation Index, m: Embedding dimension, τ: Delay, *r*: threshold, RR: Recurrence rate, DET: Determinism, LT: Average length of diagonal structures, ENTR: Shannon Entropy, LAM: Laminarity, TT: Trapping time.

Zone	RPs and CRPs	***m***	**τ**	***r***	**RR (%)**	**DET (%)**	**LT**	**ENTR**	**LAM (%)**	**TT**
ZAV	NDVI	2	8	8.67	5.00	59.19	2.81	1.23	72.32	3.01
	TEMP	2	11	8.76	4.99	40.26	2.31	0.72	52.89	2.49
	PCP	2	3	1.35	4.97	14.65	2.10	0.32	33.72	2.35
	NDVI-TEMP	2	11	9.96	5.00	48.99	2.44	0.88	58.30	2.60
	NDVI-PCP	2	8	6.34	4.99	25.96	2.26	0.64	30.46	2.45
ZMA	NDVI	2	10	7.80	4.99	63.92	2.99	1.29	77.22	3.26
	TEMP	2	11	9.00	4.99	42.02	2.35	0.76	54.35	2.60
	PCP	10	9	13.25	5.00	6.57	2.03	0.14	20.72	2.11
	NDVI-TEMP	2	11	10.53	4.99	52.42	2.51	0.96	59.65	2.63
	NDVI-PCP	10	10	26.19	4.99	24.26	2.12	0.39	27.36	2.15

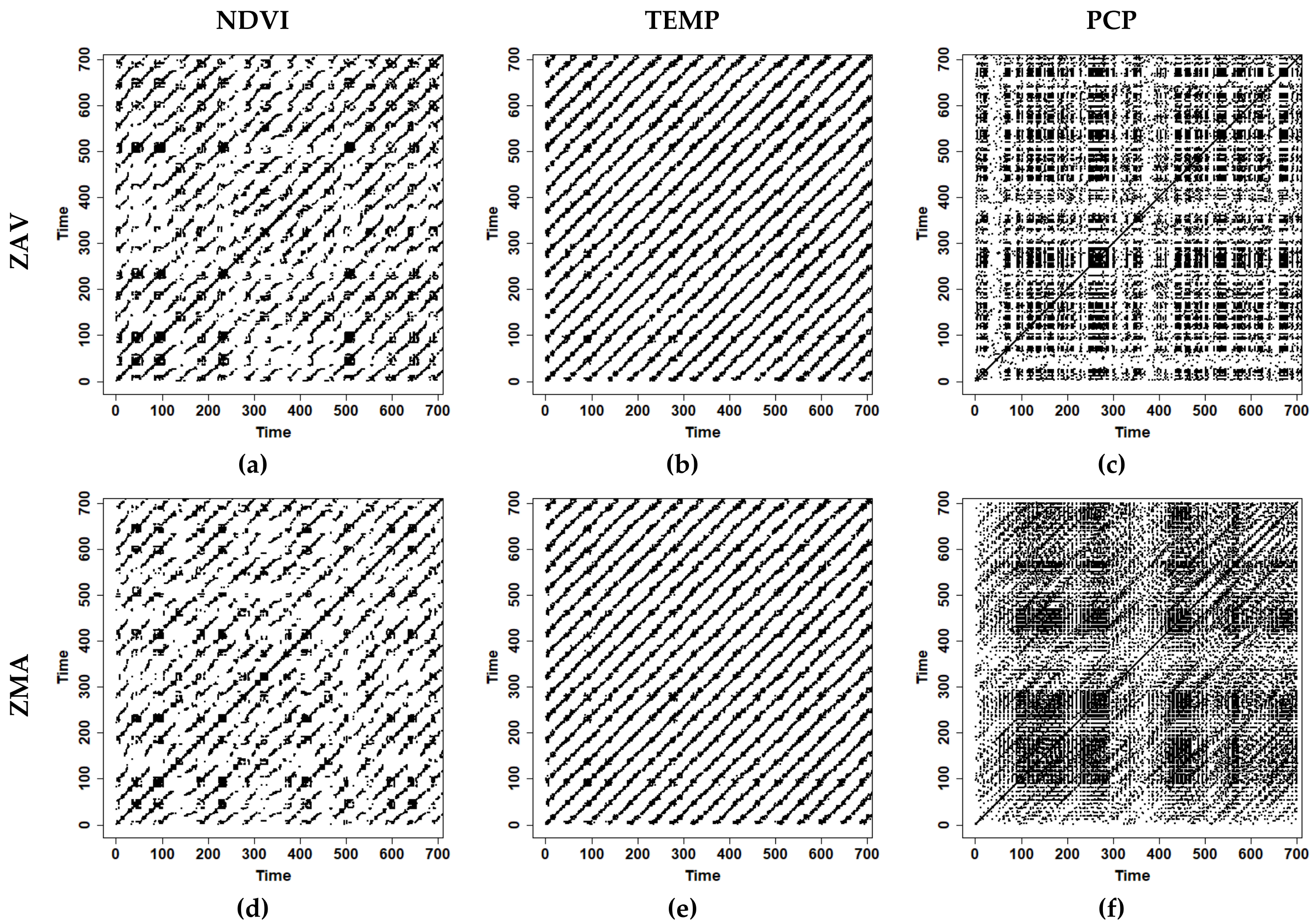

Figure S3. Optimized recurrence plots (RP) using NDVI, Average temperature (TEMP) and Accumulated precipitation (PCP) data and rescaled distance matrix for Tornadizos de Ávila (ZAV) and Soto del Real (ZMA). Time units are represented as the X and Y-axis. Each time-unit is 8-days, coincident with 8-day composed MODIS images during the study period (2002-2018). Panels **[a-c]** correspond respectively to RP of NDVI, Temperature and Accumulated Precipitation for the ZAV zone. Panels **[d-f]** correspond respectively to NDVI, Temperature and Accumulated precipitation for the ZMA zone.

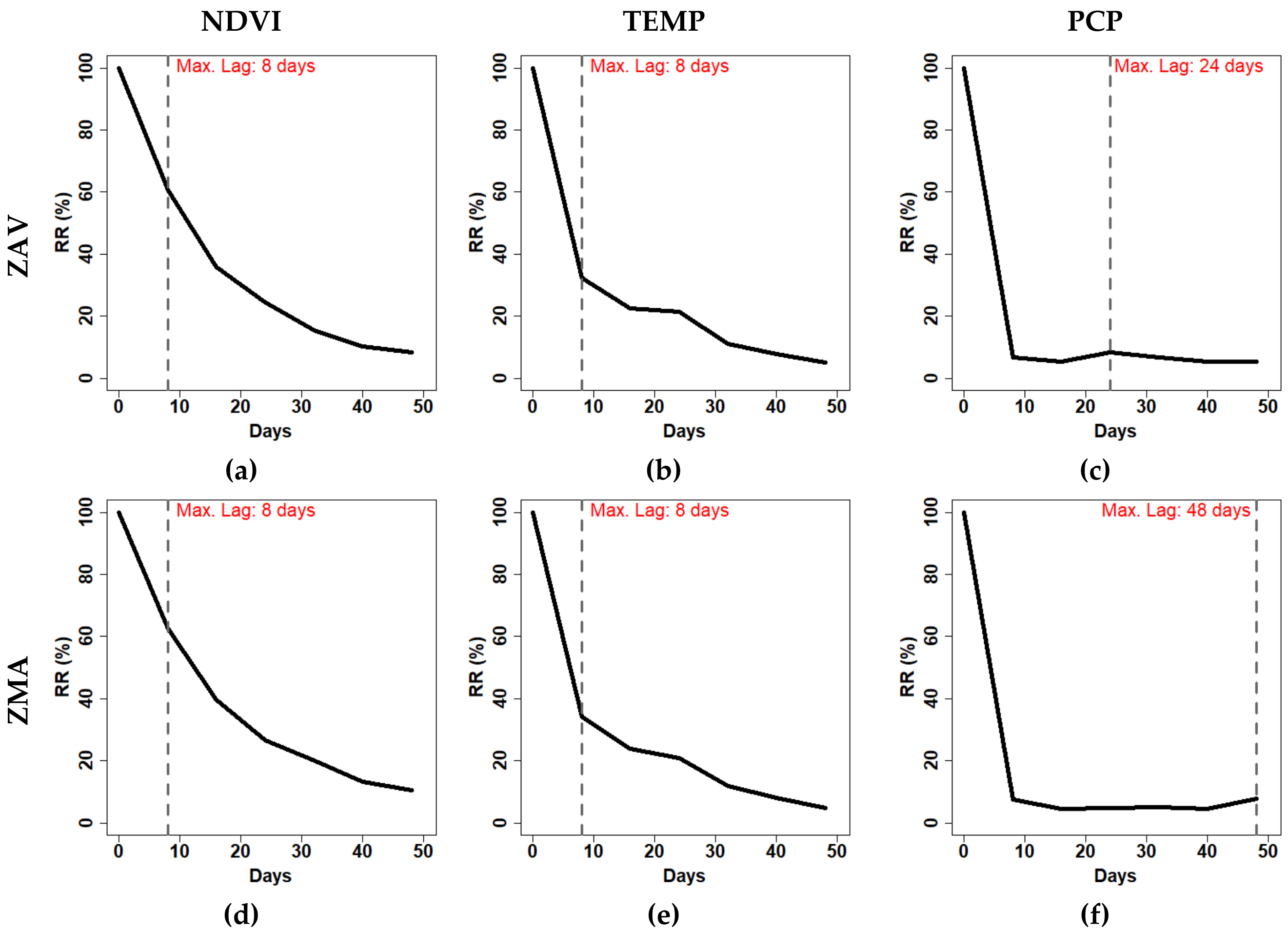

Figure S4. Diagonal-wise recurrence profile of the RPs obtained from the NDVI, Average temperature (TEMP) and accumulated precipitation (PCP) in Tornadizos de Ávila (ZAV) **[a-c]** and Soto del Real (ZMA) **[d-f]**.

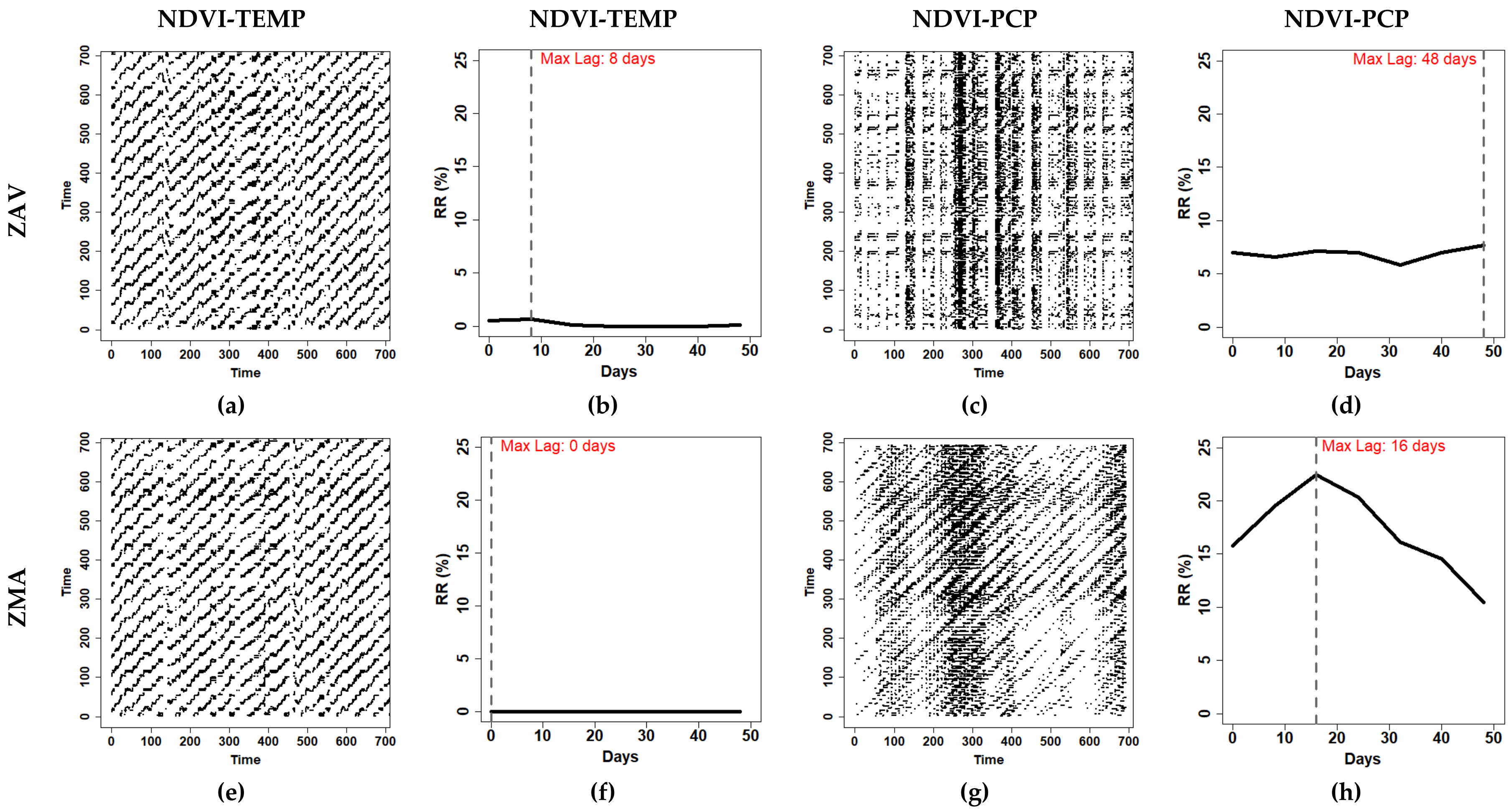

Figure S5. Optimized Cross-Recurrence Plots (CRPs) and diagonal-wise recurrence profiles between vegetation indices data (NDVI) temperature data (TEMP) and accumulated precipitation data (PCP) for Tornadizos de Ávila (ZAV) and Soto del Real (ZMA). Time units are represented as the X and Y-axis. Each time-unit is 8-days, coincident with 8-day composed MODIS images during the study period (2002-2018) in the CRPS. Lags are represented in days in the diagonal-wise recurrence profile The panels **[a,c,e,g]** represent the CRPs of NDVI-TEMP and NDVI-PCP. The panels **[b,d,f,h]** represent the diagonal-wise profile of the CRPS, respectively.
